# Biomaterials Based on Marine Resources for 3D Bioprinting Applications

**DOI:** 10.3390/md17100555

**Published:** 2019-09-28

**Authors:** Yi Zhang, Dezhi Zhou, Jianwei Chen, Xiuxiu Zhang, Xinda Li, Wenxiang Zhao, Tao Xu

**Affiliations:** 1 Department of Precision Medicine and Healthcare, Tsinghua-Berkeley Shenzhen Institute, Shenzhen 518055, China; yi-zhang16@mails.tsinghua.edu.cn (Y.Z.); chenjw17@mails.tsinghua.edu.cn (J.C.); zhangxx19@mails.tsinghua.edu.cn (X.Z.); 2 Department of Mechanical Engineering, Biomanufacturing Center, Tsinghua University, Beijing 100084, China; zhoudz17@mails.tsinghua.edu.cn (D.Z.); li-xd15@mails.tsinghua.edu.cn (X.L.); zhaowx19@mails.tsinghua.edu.cn (W.Z.)

**Keywords:** 3D bioprinting, marine resource, biomaterials, bioprinting application

## Abstract

Three-dimensional (3D) bioprinting has become a flexible tool in regenerative medicine with potential for various applications. Further development of the new 3D bioprinting field lies in suitable bioink materials with satisfied printability, mechanical integrity, and biocompatibility. Natural polymers from marine resources have been attracting increasing attention in recent years, as they are biologically active and abundant when comparing to polymers from other resources. This review focuses on research and applications of marine biomaterials for 3D bioprinting. Special attention is paid to the mechanisms, material requirements, and applications of commonly used 3D bioprinting technologies based on marine-derived resources. Commonly used marine materials for 3D bioprinting including alginate, carrageenan, chitosan, hyaluronic acid, collagen, and gelatin are also discussed, especially in regards to their advantages and applications.

## 1. Introduction

Due to the limited availability of donor tissues and organs for clinical application, more and more attention has been paid to engineered tissues. However, many challenges remain in the biofabrication of complex and heterogeneous tissues and organs for clinical translation purposes [1]. Three-dimensional (3D) bioprinting, an emerging additive manufacturing technology in which cell-laden hydrogel bioinks are deposited in a layer-by-layer fashion, shows promise for meeting the requirements of constructing complex composite living tissues, with the help of recent advances in 3D printing technology, cell biology, and materials science. Inkjet bioprinting, extrusion bioprinting, and stereolithographic 3D bioprinting are typical bioprinting methods [2]. Inkjet printing has high speed and resolution; however, it is not easy to print highly viscous solutions with this technology. Extrusion printing has lower printing resolution; however, it is easier to fabricate 3D constructs with this method due to the continuous extrusion of fiber and relatively high mechanical integrity. Although stereolithographic 3D bioprinting is not commonly used as compared to other two methods due to the difficulty of preparing printing materials and inability to print multiple types of cells, it avoids the clogging problem and can achieve high viability of printed cells [3]. Cells, biomaterials, and bio-factors are the fundamental components for 3D bioprinting to build constructs. Due to its capability of precisely fabricating complex structures, 3D bioprinting holds great promise to construct biomimetic structures for the study of disease development or tissue/organ replacement.

Material is an important factor for fabricating and designing 3D constructs that fulfill specific demands. The materials suitable for 3D bioprinting should possess properties like printability, mechanical integrity, and biocompatibility. The commonly used synthetic materials such as polycaprolactone (PCL), poly-L-lactic acid, and poly (lactic-co-glycolic acid) have high mechanical strength; however, they lack sites of cell adhesion [4]. Also, the high printing temperature and organic solvent needed to make bioink with these materials make it difficult for them to encapsulate cells. Natural materials encapsulate and interact with cells more easily; however, most of them lack the suitable mechanical strength and it is not easy to control their degree of degradation [5]. Until now, the number of materials suitable for 3D bioprinting has been limited, lowering the rate of development of this field. New resources of materials with abundant existence, high economic benefits, and unaffected by pathogen, would bring new opportunities for the development of 3D printing materials.

The ocean is such an untapped material resource for biomedical applications [6]. One example is the nacre-derived materials [7], which are abundant in the ocean and provide an economic way for developing new materials, as they are essentially the residues of seashells. At the same time, some materials from marine sources could avoid animal-derived infections compared with animal-based materials. Some new marine-based materials, such as fish-gelatin [8] and aneroin [9] have been successfully used in 3D printing and have shown their potential in tissue engineering applications. This review addresses the mechanisms, material requirements, and application of commonly used 3D bioprinting methods based on marine resources. Main marine materials used in 3D bioprinting including alginate, carrageenan, chitosan, HA, collagen and gelatin are emphasized, especially in their advantages and application. Finally, recent development, current challenges, and future prospects of marine materials applied in 3D bioprinting are also presented in this study.

## 2. 3D bioprinting Technologies

3D bioprinting technologies employ an X-Y-Z robotic system to load and read computer-aided-design files (from computed tomography scan or design software) to fabricate the tissue structure. For the fabrication of 3D cell-laden hydrogel tissue constructs, the bioink and bioprinter are key elements. In this part, the most important bioprinting technologies including inkjet, extrusion and stereolithography (SLA)-based printing are discussed.

### 2.1. Biomaterials Classification for Bioprinting

In 3D bioprinting, bioinks refer to cell-laden biomaterial solutions [10]. These biomaterials can be classified as naturally-derived (including alginate, gelatin, collagen, fibrin, HA, decellularized extracellular matrix (ECM), silk, chitosan and carrageenan [11]) or synthetic bioinks (polyethylene glycol (PEG) [12], and pluronic acid bioinks [10]).

Generally, compared to synthetic bioinks, naturally-derived ones provide better biocompatiblility in terms of cell viability and growth. Although chemical modifications of naturally-derived biomaterials change them into photo-polymerizable bioinks such as gelatin methacryloyl (GelMA), methacrylated HA (MeHA), the modification of naturally-derived bioinks is still difficult and limited [13].

### 2.2. Requirements for Biomaterials to be Used as Bioinks

**Figure 1:**
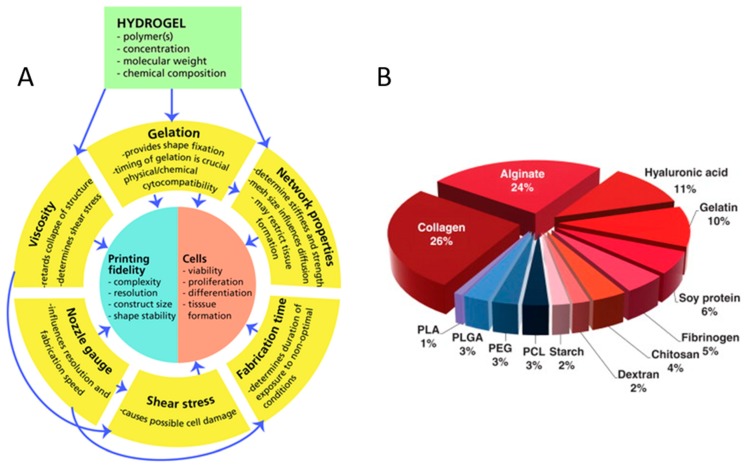
Concept map of variables and relations critical to biofabrication (**A**) with the permission of reference [14], Copyright WILEY, 2013. The polymer distributions for use as bioinks (**B**) with permission from reference [18], Copyright Elsevier, 2015.

In tissue engineering, to mimic the in-vivo micro-environment and structures, the properties of bioprinted scaffolds (Figure 1A), including cell viabilities, scaffold porosity and mechanical properties, must match those of real tissues in the body. Appropriate hydrogels must have low concentrations and high viscosity to maintain the desired shape [14], low shear stress and shear rate [15], as well as favourable shear thinning and thixotropic properties to maintain high cell viability. Furthermore, hydrogels need strong interfacial bonding between layers to maintain their stability [16]. Meanwhile, a slow degradation rate means shape retaining of the scaffold for a long time [17]. Therefore, in order to enable tissue engineering application of the printed tissue or organ, it is important that the biomaterials satisfy the following requirements for 3D bioprinting [2]:

(1) Biomaterials should be biocompatible and friendly to specific types of cells or hosts without eliciting cell death or immune response and have a positive effect on the attachment, migration, proliferation, and function of both endogenous and exogenous cells.
(2) Biomaterials need to be printable and can be accurately and precisely deposited with the desired spatial pattern and temporal control.
(3) Biomaterials need to have controlled degradation kinetics and non-toxic byproducts, as the embedded cells secrete proteases and subsequently produce ECM proteins that define the new tissue.
(4) Biomaterials-based structures need excellent mechanical properties which are essential for continued function of the construct by various crosslinking or other methods.

### 2.3. Main 3D Bioprinting Technologies

Among various 3D printing technologies, 3D bioprinters based on cell-laden bioinks often utilize inkjet bioprinting, (co-axial) extrusion printing or stereolithographic printing (Table 1). These bioprinting technologies will be discussed in detail.

#### 2.3.1. Inkjet Bioprinting

**Figure 2:**
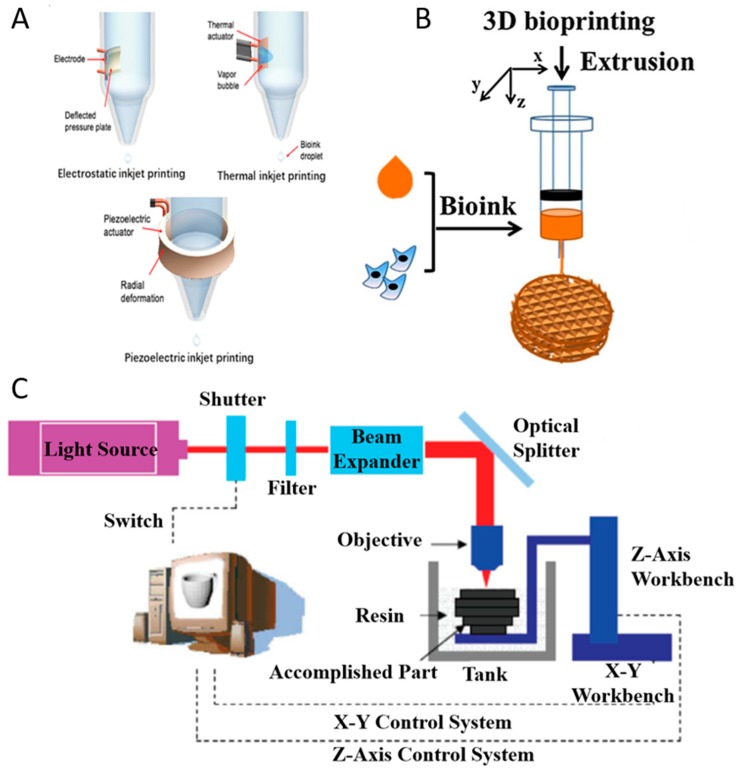
Inkjet printing technologies (**A**) with permission from reference [19], Copyright Elsevier, 2019. Extrusion printing technology (**B**) with permission from reference [27], Copyright Elsevier, 2018. Stereolithography-based bioprinting technology (**C**).

Inkjet printing can be classified as continuous-inkjet bioprinting or drop-on-demand bioprinting [19]. Compared with continuous-inkjet printer, the drop-on-demand printer ejects droplets only when it receives the signal from the controller so that it can improve the efficacy of ink utilization, and it is easy to control during the operation. Moreover, drop-on-demand bioprinting can be used for various research studies including scaffold printing (Figure 2A), cell encapsulation [20], microarrays [21], and gene transfection [22] and those may be challenging to achieve with continuous-inkjet bioprinting. Drop-on-demand bioprinting is more reliable and commonly used than continuous-inkjet bioprinting.

Drop-on-demand bioprinting manipulates the bioink to generate droplets via different physical methods. According to the driving force, drop-on-demand printers are divided into three types (Figure 2A): thermal, piezoelectric, and electrostatic forces. Inkjet printing have used various biomaterials (alginate, collagen, fibrin, etc.) as bioinks in different tissue engineering applications such as bone [23], cartilage [24], liver [25], and ocular tissue [26]. To avoid the clogging problem, the bioink used in drop-on-demand bioprinting should have low viscosity (<10 mPa·s) and cell concentration (<10^7^ cell/mL) [19]. Marine-derived biomaterials with the potential to be used as bioinks for inkjet printing include alginate and collagen [23,24].

##### Thermal Inkjet Printing

Thermal inkjet printing uses the actuator to heat the bioink in the chamber for the purpose of ejecting droplets (Figure 2A). When a voltage is applied, the heating actuator creates a vapor bubble. As the bubble expands rapidly and explodes, it produces a pressure pulse inside the chamber and the droplets are pushed out of the orifice [28]. During the printing process, the actuator raises its temperature to 300 °C [29], but cells could maintain high viability after printing due to the short period of ejection.

##### Piezoelectric Inkjet Printing

Piezoelectric inkjet printing, similar to thermal inkjet printing, also uses an actuator to eject the droplets out of the orifice. The piezoelectric inkjet printer relies on the deformation of the actuator to eject the droplets (Figure 2A). When a voltage is applied, the actuator changes its shape to deform the ink chamber which produces a pressure variation within the chamber. As the pressure overcomes the ink surface tension, the droplets are pushed out. Compared with thermal inkjet printing, piezoelectric inkjet printing has no limitation when it comes to thermally sensitive ink.

##### Electrostatic Inkjet Printing

The electrostatic inkjet printer generates droplets by manipulating the volume of the fluid chamber (Figure 2A). The chamber has two orifices: one for droplet printing and another for ink to enter the fluid chamber [30]. When a voltage is applied, the chamber changes its shape and ink enters the chamber. After the voltage is removed, the chamber regains its original shape and subsequently ejects droplets. Compared with other inkjet printing methods, electrostatic inkjet printing has a lower bioink consumption.

#### 2.3.2. Extrusion Bioprinting and Co-axial Extrusion Bioprinting

In extrusion-based bioprinting, three-dimensional constructs are formed by microfibers extruded through a nozzle. With the relative movements between the platform and the nozzle, three dimensional constructs may be deposited. The bioinks to form the microfiber should have a proper viscosity in order to ensure the connection between layers, and should be able to be crosslinked to ensure the formation of the final constructs (Figure 2B).

Extrusion-based bioprinting has been widely used to deposit various kinds of bioinks. These bioinks include hydrogel with encapsulated cells [31], ECM [5] and cell aggregates [10,32]. Among these bioinks, hydrogel with encapsulated cells is widely used for extrusion-based bioprinting. The bioinks used for extrusion-based bioprinting should have biocompatibility, printability and proper mechanical strength after crosslinking [13]. Many types of hydrogel, such as alginate [33], chitosan [34], gelatin [35], collagen [36] and HA [37] that are used in extrusion-based bioprinting can be derived from marine resources. Hydrogels that can be used in extrusion-based bioprinting should be crosslinked under certain conditions, such as specific temperature ranges [38] or ionic conditions [39]. Gelatin can be crosslinked at a low temperature and alginate can be crosslinked with calcium ions. Meanwhile, alginate and gelatin both have shear thinning properties, making them suitable for extrusion-based bioprinting [40]. When the strain rate increases, a decreased viscosity of shear thinning bioinks can protect encapsulated cells. Moreover, shear thinning allows for smooth extrusion of viscous bioink through the nozzle to improve the resolution by limiting the entanglement of chains [13]. Chitosan has a slow gelation time and low mechanical properties [41], and thus it is usually blended with other hydrogels for proper printability. HA also has low mechanical properties and a short degradation time [42]; thus, modification is usually needed [43].

Furthermore, with a coaxial nozzle, these biomaterials can be printed coaxially [44]. A core-shell microfiber can be created in this manner. The core can possess either good mechanical strength [44] or poor mechanical strength [45]. The shell is always made of alginate since it has proper mechanical strength [45,46]. With protection from the shell, the core may be designed with good biocompatibility and encapsulate a large amount of cells. Ouyang et al. have done a series of studies using biomaterials from marine resources, mainly alginate, to print cells [40,47]. Using proper bioink parameters and bioprinting parameters based on the cell type selected is pivotal in keeping the structural integrity and cell viability in extrusion-based printing [48]. Therefore, proper bioink should be selected, and printing parameters should be carefully adjusted to fit the requirement of the cells being printed.

#### 2.3.3. Stereolithographic 3D Bioprinting

The SLA-based bioprinting technology utilizes the spatially-controlled irradiation of light or laser to solidify a geometrically two-dimensional (2D) pattern layered through selective photopolymerization in the bioink reservoir (Figure 2C). The 3D structure can be consecutively built on 2D patterned layers in a “layer-by-layer” fashion, and the uncured bioink can be easily removed from the final product.

SLA is a solid freeform technology that was introduced in the late 1980s which has the highest fabrication accuracy, and the number of materials that can be processed with SLA is increasing. SLA is particularly versatile with respect to the freedom of designing structures and the scales at which these can be built: sub-micron sized structures, as well as decimetre-sized objects have been fabricated. In the biomedical field, these developments have led to the fabrication of patient-specific models for mould-assisted implant fabrication [49]. More recently, biodegradable materials have been developed for the preparation of medical implants, such as tissue engineering scaffolds, by SLA [50].

Kinetics of the curing reactions occuring during polymerization is critical. For example, ultraviolet (UV) absorbers can be added to the resin to control the depth of polymerization [51]. In addition, materials must have photocurable moieties for photocrosslinking. The advantages of SLA are the ability to create complex shapes with internal architecture, ease of removal of unpolymerized resin, and extremely high feature resolution (~1.2 um) [52]. The main disadvantage of SLA is the scarcity of biocompatible resins with proper SLA processing properties. Additional challenges are the use of photointiators and radicals which may be cytotoxic (with long processing times), entrapment of unreacted monomer and residual photoinitiator, and an inability to create compositional gradients along horizontal planes. Photopolymerized resin also has poor mechanical properties that are needed for hard tissue engineering. However, natural biomaterials with some modification (eg. acetylation, such as GelMA, and MeHA) showed the potential of SLA-based bioprinting for tissue engineering applications [53,54].

## 3. Marine-Derived Biomaterials for 3D Bioprinting

In 3D bioprinting, as bioinks contain living cells, biomaterials are key components. These biomaterials are compatible with biological materials (cells, cell aggregates, microcarriers, etc). In addition, they are suitable for the printing process and provide the relevant mechanical and functional properties.

Marine organisms are natural source reservoirs of various biopolymers with diverse biological cues (Table 2). However, efficient use of marine organisms is still lacking. Due to marine-derived biopolymer diversity and simplicity of the isolation processes [55,56,57], marine biomaterials for 3D bioprinting applications have received rapidly increasing attention. Many marine-derived biomaterials are selected as bioinks in 3D bioprinting (Figure 1B), including not only the widely used alginate, chitosan [10], and carrageenan [58], but also HA, collagen, and gelatin [5,8,55]. Marine-derived biomaterials for 3D bioprinting are naturally-derived bioinks, showing necessary properties and advantages for tissue engineering. Compared to mammalian resources, marine natural resources do not carry risks of transmissible diseases and are unaffected by religious restrictions. Therefore, interest in biomaterials for 3D bioprinting has changed into marine resources because they enable high production at a low cost [56]. At the same time, compared to synthetic hydrogels, marine-derived natural hydrogels have many advantages, such as sufficient biological cues, low immune response, and excellent biocompatibility for tissue engineering applications.

## 4. Characteristics and Potentials of Main Marine-Derived Biomaterials for 3D Bioprinting

Marine-derived compounds have potential in a wide range of applications because of their suitable chemical structures and functionalities. Among various marine-origin macromolecules, alginate, carrageenan, chitosan, HA, collagen, and gelatin in particular have emerged as widely used biomaterials for 3D bioprinting of regenerative medicine in recent years (Table 2). In fact, marine biomaterials satisfy most requirements of 3D bioprinting, such as printability, mechanical integrity, and biocompatibility.

### 4.1. Commonly used Marine-Derived Biomaterials as Bioinks

#### 4.1.1. Alginate

**Figure 3:**
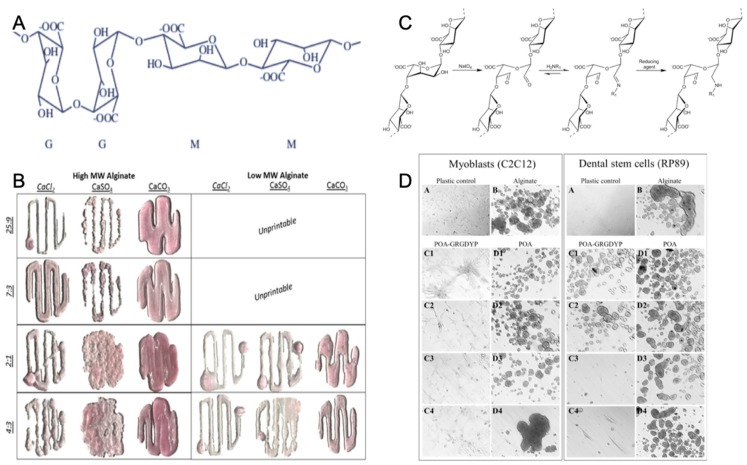
Chemical structure of alginate (**A**) with permission from reference [69], Copyright Elsevier, 2003. The gelation and printability of different molecular weight alginate bioinks with Ca^2+^ (**B**) with permission from reference [61], Copyright Springer Nature, 2017. The coupling strategy of the tripeptide arginine-glycine-aspartic acid sequence with alginate (**C**) and the increased adherence of the cells to modified alginate (**D**) with permission from reference [70], Copyright Elsevier, 2015.

One of the most representative polysaccharides in marine environment is alginate [56]. In general, alginate, which is a linear anionic polysaccharide copolymer of (1–4)-linked β-mannuronic acid (M) and α-guluronic acid (G) monomers (Figure 3A), is derived primarily from brown seaweed and also from bacteria [67]. Commercial alginate extraction started in 1929 and has been developed to obtain alginate with controlled molecular weight (MW), which acts as source of biomaterials with specific physico-chemical properties for tissue engineering [68].

Gelation is an important characteristic of alginates. Today, alginate is widely used as a gelling agent for pharmaceuticals, tissue engineering, biomedicine, and the food industry [71,72,73]. Alginate hydrogel, which is a naturally-derived polymer, has a similar physical structure to the native ECM, gentle gelling kinetics, good biodegradability, excellent biocompatibility, and low toxicity [74]. Alginates can form a hydrogel polymer with well-known properties in the presence of divalent counterions (Ca, Ba, Sr) (Figure 3B). Calcium chloride (CaCl_2_) is a typical ionic crosslinker because of rapid gelation with its high solubility in aqueous solutions [75]. The ionic crosslinker can distinctly influence the printability, mechanical integrity, and the degradation rate of alginate-based hydrogel, leading to low degradability [61].

The alginate hydrogel has the potential to become a scaffold material for tissue engineering, since it offers many advantages over synthetic polymers for this application. Alginate is abundantly available in marine environments and can be obtained with low cost, and it forms hydrogel under relatively mild conditions (pH and temperature). Alginate is also nontoxic, biocompatible, and biodegradable [76]. Tissue engineering applications of alginate hydrogel have received increasing interest. Alginate hydrogel not only has mechanical integrity to produce scaffolds [77], but also is nontoxic and biocompatible to easily encapsulate cells as bioink during the hydrogel formation process [61,72,78]. In addition, alginate has plasticity of molecular structure which benefits 3D bioprinting by adding the cell attachment sites. Alginate molecules may enhance the interaction with cells by incorporating arginine-glycine-aspartic acid (RGD) sequences along the chain of macromolecular structure (Figure 3C,D) [70,79].

#### 4.1.2. Carrageenan

**Figure 4:**
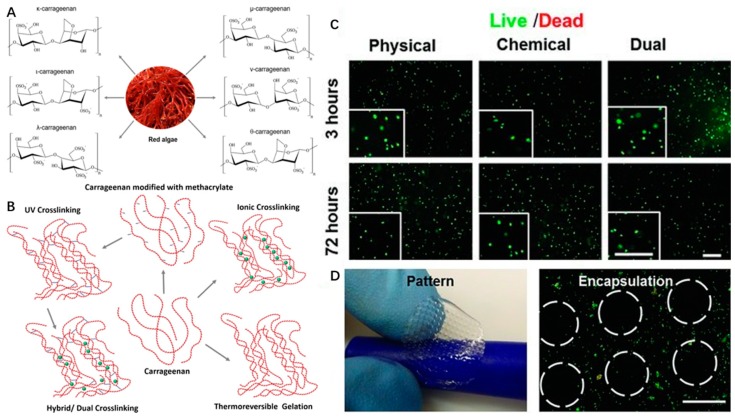
Chemical structures of different types (**A**) and different crosslinking mechanisms (**B**) of carrageenan with the permission of reference [58], Copyright Elsevier, 2018. The live/dead images (**C**) and bioprinted scaffolds (**D**) of encapsulated NIH-3T3 cells in MA-κ CA (Methacrylated Kappa-Carrageenan, 5%) hydrogel with the permission of reference [81], Copyright WILEY, 2013.

Carrageenan is a water-soluble anionic sulfated polysaccharide derived from the marine red algae by alkali extraction. Carrageenan is a galactan, and it consists of repeated sequences of b-d-galactose and a-d-galactose with variable proportions of sulfate groups. Seaweed-derived carrageenan can be divided into six basic forms based on the position and number of sulfate groups: κ-(kappa), ι-(iota), λ-(lambda), μ-(Mu), ν-(Nu) and θ-(Theta)-carrageenan (Figure 4A) [80].

Carrageenan type, molecular weight, concentration, and temperature have effects on the viscosity of carrageenan gels. Among the above types of CRGs, κ and ι types of carrageenan, which can form hydrogel at low temperature, and low salt concentrations are commercially popular because of their viscoelastic and gelling properties (Figure 4B). For example, κ-carrageenan can coil to helix transition and helical aggregation to form thermotropic and ionotropic hydrogels at low temperature (5 °C) and in the presence of cations (K^+^, Ca^2+^) [80]. It is known that the viscosity of carrageenan gels will decrease with increasing temperature.

However, carrageenan-based hydrogels are brittle in nature with high swelling ratios and poor mechanical stability under physiological conditions [82]. The degradation mechanism of carrageenan hydrogels is similar to that of alginate hydrogels, where ion exchange with the surrounding medium causes mechanical weakness [83]. Two types of strategies have been adapted to overcome this limitation. First, to improve mechanical properties and printability of carrageenan hydrogels, double network hydrogels can be formed by combining carrageenan with other components. Hydrogels fabricated with this approach showed super strong interface bonding [84,85]. In addition, various chemical modifications of the carrageenan backbone with hydroxyl/sulphate groups, such as oversulphated, acetylated, phosphorylated [86], oxidized [87], carboxmethylated [88], and methacrylated [81] modifications, also have been used to overcome this weakness [89].

Therefore, carrageenan-based hydrogels can be formed by four different crosslinking mechanisms, including thermoreversible gelation, ionic crosslinking (in presence of monovalent or divalent ions), UV crosslinking (modification of carrageenan backbone with methacrylate groups), or dual crosslinking (both UV and ionic crosslinking) (Figure 4B).

Carrageenan shows similar tissue composition to mammalian glycosaminoglycans of ECM [90]. Therefore, the thermogelation properties and excellent biocompatibility of κ-carrageenan hydrogels have been used to improve rheological behaviors of hydrogel and precise fabrication of the cell-laden scaffolds based on alginate/carrageenan hydrogels in 3D bioprinting [91]. κ-carrageenan can also be used as a biological binder in the printing of the tissue scaffold with its proven biocompatibility [85]. In addition, methacrylated carrageenan (MA-κ-carrageenan) underwent gelation upon the consecutive crosslinking procedures (UV and KCl) and showed good printability of scaffolds with encapsulated cells. Human mesenchymal stem cells (hMSCs) within MA-κ-carrageenan hydrogel presented high viability (∼75%) for long time periods (up to 21 days) (Figure 4C,D) [81]. These chemical modifications of carrageenan endow them with improved physio-chemical properties, new specific functionalities and features, such as a capability for photocrosslinking for SLA-based 3D bioprinting in tissue engineering applications.

#### 4.1.3. Chitosan

**Figure 5:**
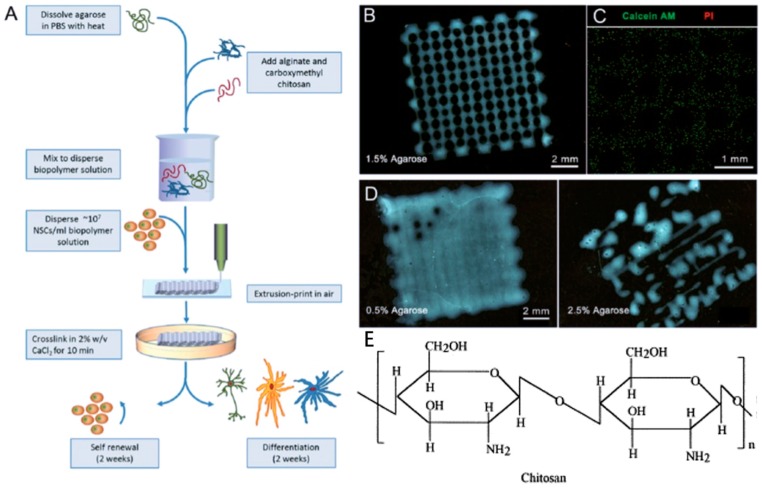
The generation of 3D micro neural scaffold with human neural stem cell-laden in chitosan hydrogel (**A**–**D**) with the permission of reference [95], Copyright WILEY, 2016. Chemical structures of chitosan (**E**) with the permission of reference [62], Copyright Elsevier, 2000.

Chitosan, a cationic polysaccharide, is the deacetylated derivative of chitin which is the second most abundant natural polymer after cellulose [62,92,93]. Among the various polysaccharides obtained from the marine environment, chitosan is also one of the most abundant polysaccharides. It is the main structural component in the exoskeleton of various marine invertebrates [56]. Chitosan is formed by d-glucosamine (70–90%) and *N*-acetyl-d-glucosamine (10–30%) units, linked by β-1,4 glycosidic bonds (Figure 5E) [62]. The deacetylation degree of chitosan is related to the ratio between the two units [94].

Unlike chitin with poor solubility, chitosan with high deacetylation degree (>50%) can dissolve in acidic aqueous solutions [96]. Because the free amino groups of chitosan are protonated in diluted acids, chitosan becomes fully soluble when pH < 5 [97]. Therefore, chitosan-based hydrogel is pH-sensitive. For example, viscosity could increase with high pH by addition of NaOH or KOH coagulation solutions [98].

Chitosan is a bioactive polymer with favorable properties such as biocompatibility, bioactivity, high mechanical strength, easy modification, and biodegradability. It is used in a variety of applications such as neural tissue engineering (Figure 5A–D) [62]. In addition, chitosan has antimicrobial property due to charge interaction with the membranes of microorganisms [99,100,101]. Chitosan-based hydrogel as a bio-ink for 3D bioprinting in bone tissue engineering clearly demonstrated the feasibility and printability of chitosan as a bio-printing solution, and cells encapsulated within chitosan-based hydrogels had mineralized and differentiated osteogenically after 21 days of culture [102]. The amino groups of chitosan can be chemically modified to obtain more derivatives with satisfactory solubility, and mechanical and biological properties, which can be used for specific purposes [5,103]. But in general, thermo-mechanical processing is not suitable for chitosan because it is not thermoplastic and degrades before melting [104]. In order to avoid degradation of chitosan, the processing temperature must be controlled.

### 4.2. Selective Use of Marine-Derived Biomaterials as Bioinks

#### 4.2.1. HA

**Figure 6:**
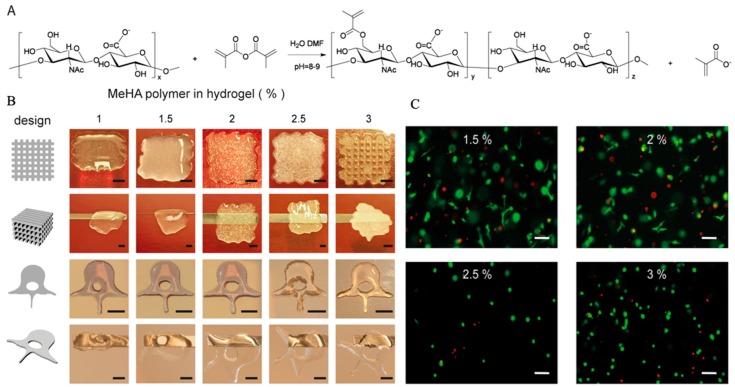
Chemical primary structure and the esterification mechanism of HA (**A**), hydrogel printability (**B**) and cell viability (**C**) in HA hydrogel with the permission of reference [64], Copyright, Poldervaart et al., 2017.

HA is a naturally occurring non-sulfated cationic glycosaminoglycan that was first extracted from bovine vitreous by Karl Meyer and John Palmers in 1934 [105]. But this is limited by the possibility of transmissible diseases and religious restrictions, so a large amount of HA has been obtained from the marine environment, mainly in cartilaginous fishes and in vitreous humor of different fishes [55,56]. HA is a polymer compound which consists of alternating disaccharide units of *N*-acetylglucosamine and glucuronic acid, linked by β-1,3 and β-1,4 glycosidic bonds (Figure 6A) [106].

It is well known that the hydrophobic and hydrogen bonding interactions, countering electrostatic repulsion, enable large numbers of HA molecules to aggregate, leading to the formation of molecular networks (matrices) [63]. However, the stability of the matrix structure of HA appeared to be marginal in physiological solutions [107]. Therefore, indirect HA-based hydrogel is used in 3D bioprinting applications by esterification of the primary hydroxyl groups and photocrosslinking [108]. When functionalized with methacrylate groups (Figure 6A), MeHA could form hydrogel networks by the addition of a photo initiator, leading to polymerization upon UV-exposure.

HA is an ECM component and its physiological functions include maintaining the viscoelasticity of connective tissue fluids, controlling tissue hydration and water transportation, and assembling supramolecular proteoglycans in the ECM [109,110,111]. Because of the above advantages, HA is widely applied in the field of biomedicine with few reported adverse effects [112,113]. In 3D bioprinting, most previous studies simply used HA as an additive component to improve cell growth, as it plays an important role in the mediation of cell physiological functions via interaction with binding proteins and cell surface receptors [63], regardless of instability of HA networks in solution [114,115]. When compared to plain HA hydrogels, modified MeHA hydrogels have been shown to be more resistant to degradation, while maintaining good biocompatibility. In addition, photocrosslinking-enabled MeHA hydrogels could be applied as cell-laden bioink to print tissue scaffolds by crosslinking with UV light directly after gel deposition (Figure 6B) [64]. When encapsulated in MeHA-based hydrogels with increased mechanical stiffness and long-term stability, the viability of hMSCs remained at 64.4% after 21 days of culture, and osteogenic differentiation of hMSCs occurred spontaneously in hydrogels with high concentrations of MeHA polymer, in the absence of additional osteogenic stimuli (Figure 6C) [64].

#### 4.2.2. Collagen

**Figure 7:**
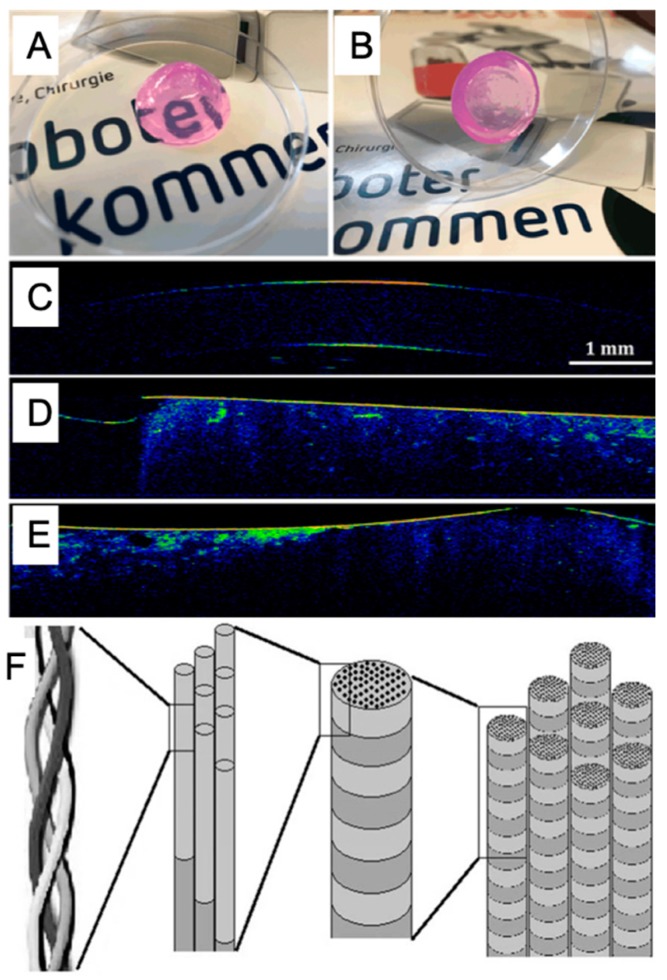
The printability (**A**,**B**) and cell viability (**C**–**E**) in collagen hydrogel with the permission of reference [118], Copyright WILEY, 2019. Chemical structures of collagen (F) with the permission of reference [119], Copyright Creative Commons Attribution License (CC BY 3.0).

Collagen is a natural protein polymer with the structure of a triple helix by three extended protein chains that wrap around one another (Figure 7F) [116]. These protein chains contain cell adherence sites based on integrin binding motifs of RGD residues. Moreover, collagen is a unique protein with abundant non-polar amino acids, such as Glycine–Gly (30%), Alanine–Ala (10%) and Proline–Pro (10%), and significant presence of Hydroxyproline–Hyp, leading to an elegant structural motif [117].

Molecular structure, MW, and temperature have effects on the characteristics of collagen and determine the viscosity and formation of collagen-based hydrogels. For example, collagen type I hydrogel is temperature-sensitive, and when the environmental temperature increases to 20–28 °C, collagen molecules could aggregate to form thermotropic hydrogel [65].

Collagen is part of the ECM and mainly found in hard and soft connective tissues of the skins and bones of animals. Collagen has the advantages of biodegradability, biocompatibility and low immunogenicity, so it is broadly used in 3D bioprinting for tissue engineering applications [116,119,120]. Since hydrogel based only on collagen presents weak mechanical properties, collagen has been modified by esterification or blended with other materials like alginate and gelatin as bioink to fabricate tissue models or repair structures. Recently, collagen has been applied in cornea fabrication [118,121], skin healing [122,123], and thyroid gland [124] applications by 3D bioprinting (Figure 7A–E).

Collagen is the most abundant protein presents in the human body, especially in connective tissues. Currently, commercial collagen is generally sourced from mammalian-derived products including pig and cattle hide and bone [125]. However, collagen proteins from mammalian tissues have a potential risk of transmission of diseases such as bovine spongiform encephalopathy, foot and mouth diseases, and avian influenza [126,127]. Therefore, it is critical to find a source of collagen that is appropriate for medical applications. Several studies indicated that marine collagen is an ideal substitute because fish by-products, such as skin, scale, and bone, are abundant sources of collagen [128,129,130]. Compared to land-based mammalian collagen, marine collagen has unique advantages including its large amount, low melting point, low viscosity in solution, good water solubility, and no risk of transmitted diseases [127]. To date, collagen has been found in various marine species including corals, sponges, sea urchin, salmon, jellyfish, mollusk, and coralline red algae [131].

#### 4.2.3. Gelatin

**Figure 8:**
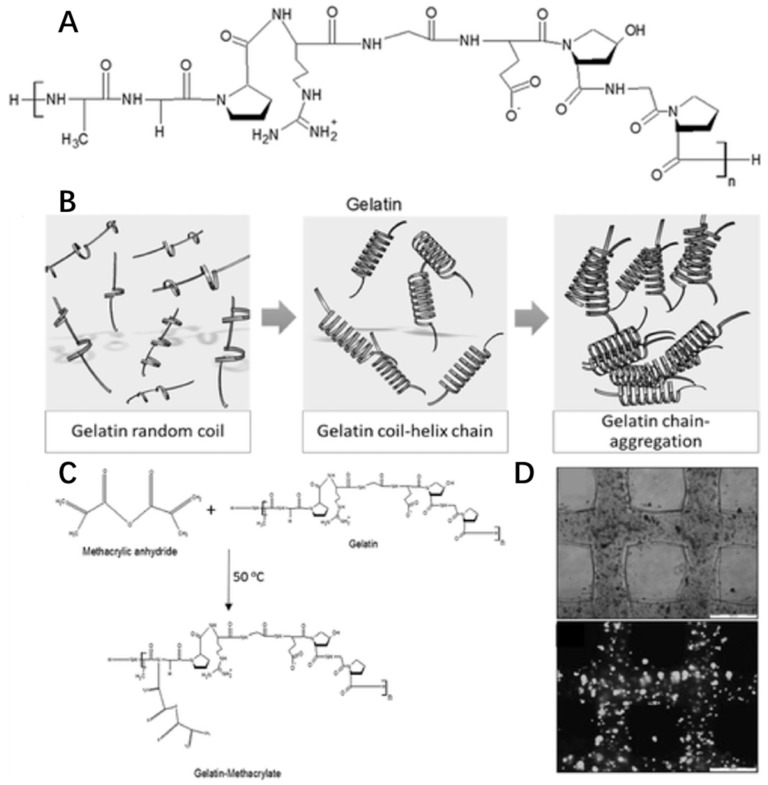
Chemical structures of gelatin (**A**), gelation (**B**), modification (**C**) and cell viability (**D**) in gelatin hydrogel with the permission of reference [132], Copyright WILEY, 2016.

Gelatin is a natural cationic biopolymer derived from collagen. In fact, gelatin and collagen are similarly structured macromolecules since gelatin is the partially hydrolyzed form of collagen (Figure 8A). However, gelatin has an opposite thermo-responsive property from collagen. The viscosity of gelatin solution will increase with decreasing temperature (<35 °C). When below 35 °C, gelatin forms a random coil structure to produce a helix leading to molecular chain aggregation for gelation (Figure 8B) [66].

Like collagen, gelatin-based hydrogel has cell adherence sites based on integrin binding motifs of RGD residues. Gelatin also shows great biodegradability, low antigenicity and biocompatibility, so it is used as a bioink material in 3D bioprinting for cell culture, such as liver tissue [133,134], cartilage tissue [135], and muscle tissue [136]. Due to its weak strength and water-solubility above 35 °C, gelatin could be modified with a methacrylate group, creating the photocrosslink-able GelMA (Figure 8C) [137] used in extrusion printing [53], inkjet printing [138] and SLA-based printing [139]. In addition, by using commonly used extrusion printing, GelMA scaffolds could be designed to have a 100% interconnected pore network in the gelatin concentration range of 10–20 w/v% and mechanically stable cell-laden gelatin methacrylamide scaffolds with high cell viability (>97%) could be printed (Figure 8D) [53].

Similar to collagen, gelatin from marine sources is free from risk of disease transmission and religious restrictions associated with the use of that obtained from mammalian sources. Thus, it is a promising alternative to mammalian gelatin [140,141].

## 5. 3D Bioprinting Applications Based on Marine-Derived Bioinks

With the development of printing technology, materials science, and stem cell biology, 3D bioprinting has made it possible to generate functional tissues in vitro [1]. Suitable biomaterials play a critical role in 3D bioprinting for tissue engineering and regenerative medicine. Marine-derived natural biomaterials fulfill the requirements of successful 3D printing based on cell-laden bioinks [56] and have been widely used in medical applications including tissue or organ regeneration (Table 3), cell therapy, and drug screening (Table 4).

### 5.1. Tissue Engineering

With the need of donor tissue or organs and the development of tissue engineering and regenerative medicine, 3D bioprinting makes it possible to fabricate 3D tissues with complex geometries of personalized consumer product in future with rapid prototyping and manufacturing technologies. By using natural marine-derived biomaterials, several tissues have already been explored for 3D bioprinting.

Alginate hydrogel is a widely used biomaterial due to its excellent performance in bioprinting for various tissue engineering applications, including bone, neural, liver, skin, and so on (Table 3). For example, in 2007, Coppi and colleagues embedded human amniotic fluid-derived stem (hAFS) cells in an alginate/collagen scaffold by thermal inkjet printing and demonstrated bone formation induced from hAFS cells [23]. Neural mini-tissue [95] and aortic valve conduits [71] had been printed by extrusion bioprinting technology and they expressed characteristic functional molecular. In addition, carrageenan, chitosan, and f-gelatin have become suitable in 3D bioprinting. Carrageenan increased the compressive strength of collagen-hydroxyapatite composite gel [142]. This improvement in mechanical properties justifies carrageenan as an efficient bone repair material. Moreover, to overcome the difficulty of fabricating functional engineered implants with patient-specific sizes and low immunogenicity, Cuidi et al. [143] developed a printable and biocompatible hydrogel which contains hydroxybutyl chitosan and oxidized chondroitin sulfate. Human adipose-derived mesenchymal stem cells (hADMSCs) encapsulated in the 3D hydrogel showed high viability. The results demonstrated that hydrogels triggered low level of inflammatory genes expression of macrophage in vitro and feeble inflammatory responses in vivo, and have the potential for cartilage engineering. Particularly, f-gelatin showed distinct potential for tissue engineering. For example, Jeong and colleagues studied the differences between GelMA from mammalian sources and fish-derived gelatin. Compared to porcine GelMA, fish GelMA had a higher degradation rate and lower melting and gelling points. Additionally, cells retained high viability until 24 h after encapsulation [144]. Zhang and colleagues used this type of f-GelMA to develop a marine-based interpenetrating polymer network hydrogel composed of fish-gelatin and alginate [8]. Besides a desirable swelling ratio, this hydrogel possessed a strong mechanical strength that can reach 130 kPa. Moreover, this hydrogel had a desirable degradation rate in both Dulbecco’s Phosphate-Buffered Saline and collagenase type II. In the morphology and cell viability study after bioprinting, the f-GelMA showed a clear structure and cells encapsulated had high viability.

#### Marine Bioinks-Based 3D Bioprinting for Autologous Tissue Replacement

##### Adipose Tissue

The biofabrication of autologous tissue substitutes provide patients with new hopes given the shortage of donors. Adipose tissue has the ability to differentiate down the mesenchymal or non-mesenchymal pathways to offer future perspectives for tissue reconstruction therapies [145]. Marine bioinks-based bioprinting technology has shown remarkable potential in adipose tissue engineering. For example, hADMSCs-laden alginate and blood plasma hydrogel scaffolds were constructed by bioprinting. Adipogenic markers that present in natural adipose tissue could be verified in the 3D grafts to resemble cell lineages [146].

##### Aortic Valve Construct

Aortic valve disease is a significant cause of morbidity and mortality. This disease could be cured by replacing the diseased valve with a prosthetic one, but current prosthetic devices are inadequate for young adults and growing children [147]. Tissue engineered living aortic valve scaffolds have the potential for tissue regeneration. Several studies have shown the potential of the bioprinted scaffolds for development of aortic valve construct. Alginate and gelatin-based hydrogel valve conduits encapsulating aortic root sinus smooth muscle cells (SMC) and aortic valve leaflet interstitial cells (VIC) were fabricated using an extrusion printer where cells were viable (81.4 ± 3.4% for SMC and 83.2 ± 4.0% for VIC) and elevated muscle actin and vimentin expression for aortic valve tissue were observed [71]. Besides, crosslinking parameters showed the effects on viability of heart valve cell types in GelMA, PEG-diacrylate 3350 (PEGDA3350) and alginate-based 3D bioprinted scaffolds. The results showed that the highest viability achieved was >93% for SMC, VIC and hADMSCs, and intracellular oxidative stress did not improve cell viability [148].

##### Bone Tissue

**Figure 9:**
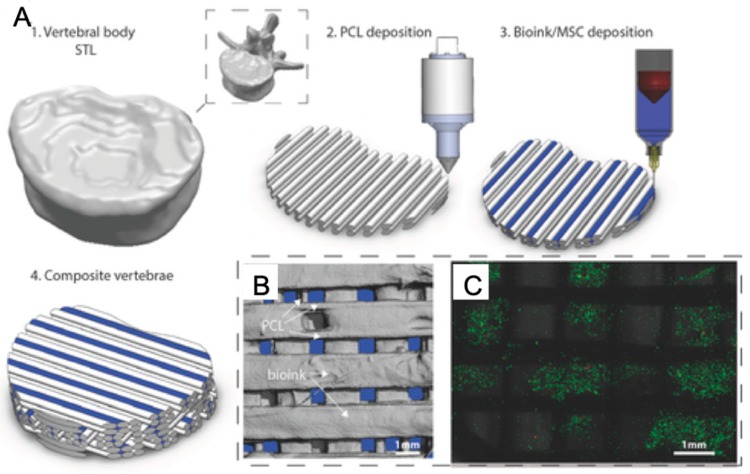
3D Bioprinting of bone tissue (**A**: Description of multi-tool 3D bioprinting process. **B**: The distribution of bioink and polycaprolactone (PCL) within tissue scaffold. **C**: Live/dead images of cells within the bioink.) with permission from reference [158], Copyright WILEY, 2016.

Bioprinting technology has shown promising developments in bone tissue, which is a complex composite tissue and needed for bone injury patients (Figure 9). In an earlier study, natural polymer-based hydrogels such as alginate, collagen, and gelatin, were mixed with bone-related stem cells and bioprinted to form 3D bone scaffolds where differentiated osteogenic lineage cells formed tissue-engineered bone and expressed elevated bone morphogenetic protein 2 (BMP-2) [23,149]. Later, hydrogel composites were improved by adding bioactive materials. For example, biosilica as a morphogenetically active matrix and human osteogenic sarcoma (SaOS-2) cells were embedded into a Na-alginate-based hydrogel for 3D bioprinting of bone tissue constructs and SaOS-2 cells presented increased growth and higher expression of the genes encoding the cytokine BMP-2 [150]. In another research study, bone-related alginate hydrogels also showed higher viability and activity by adding polylactic acid (PLA) [151], nanosilicate clay [152], hydroxyapatite [153,154,155], and cellulose [156]. Besides, collagen, alginate, and fibrin bioink-based reactive jet bioprinting offers a combination of higher deposition rate, cell density, and cell viability than some other bioprinting technologies [157]. On the other hand, chitosan and hydroxyapatite-based hydrogel tissue constructs blended with pre-osteoblast cells were stable, and its printability is superior to those made from alginate, which is the most widely used solution preferred in bioprinting systems, and the most preferred one in terms of cell proliferation and differentiation [102].

##### Cardiac Tissue

Myocardial infarction (MI) leads to a high death rate in cardiovascular diseases. Due to the progressive loss of cardiomyocytes from MI, there is a tremendous demand for new cardiomyocytes for MI treatments [159]. Recently, cardiac tissue engineering has shown promising strategies for MI repair. Zhang and colleagues showed a novel hybrid strategy based on 3D bioprinting. First, endothelial cells were directly bioprinted within microfibrous alginate and GelMA hydrogel scaffolds to form a layer of confluent endothelium. Then, cardiomyocytes were seeded on 3D endothelial scaffolds to generate human cardiomyocytes with the ability of contraction [160]. In particular, cardiac patches should be electrically conductive, mechanically robust, and biologically active. For example, in another study, a nanoreinforced hybrid way for cardiac patch bioprinting based on the combination of functionalized carbon nanotubes, methacrylated collagen, and alginate matrix showed improved electrical, mechanical, and biological behaviors for prevascularized hybrid cardiac patches [161]. For biological activity, Maiullari and colleagues presented heterogeneous, multi-cellular constructs based on alginate and PEG-fibrinogen bioinks containing human umbilical vein endothelial cells (HUVECs) and induced pluripotent stem cell-derived cardiomyocytes (iPSC-CMs) through a co-axial nozzle extruder [162]. However, although alginate bioink was used to bioprint cardiac constructs with varying architectures, the electrical/mechanical behavior of alginate implants was bioprinting pattern-dependent [163].

##### Cartilage Tissue

Cartilage tissue engineering by 3D bioprinting has received increasing attention due to the limited self-repair capability of cartilage. Natural polymers from marine sources including alginate, carrageenan and chitosan have all been intensively studied as possible bioinks [164,165,166,167,168,169,170]. For example, alginate-based bioink laden with cartilage progenitor cells (CPCs) was fabricated into hollow filament scaffolds by using a coaxial extruder and these tissue constructs yielded a relatively higher expression of cartilage-specific genes compared with monolayer cultured CPCs [166]. Due to limitations of single-component hydrogel systems, more interest for cartilage tissue engineering has been paid to biocompatible materials with good mechanical or biological properties. Such materials include cellulose [72,171,172,173], hydroxyapatite [157,174], nanosilicates [82], chondroitin sulfate [143], and cartilage-ECM (cECM) [175]. Rathan and his colleagues developed cECM-functionalized alginate bioink for the bioprinting of cartilage tissues with robust chondrogenesis property [175].

##### Dental Tissue

Due to the intricate 3D features and geometrically-controlled mechanical and biological functions of dental tissues, the regeneration of various dental structures has been addressed only to a very limited extend. However, 3D bioprinting technology has shown promising potential for biofabrication of dental tissue constructs. For example, bioprinted tissue with bioink consists of alginate, stem cells and dentin ECM showed significant odontogenic differentiation of stem cells and natural odontogenic capacity for regenerative dentistry [176]. Also, 3D-printed alginate and gelatin scaffold containing human dental pulp stem cells (hDPSCs) promoted osteogenic and odontoblastic differentiation of hDPSCs with the enhanced formation of bone-like nodules and positive alkaline phosphatase staining [177].

##### Liver Tissue

For patients suffering from end-stage liver disease, 3D bioprinting technology provides hope for its ability to biofabricate liver tissue. For example, 3D bioprinted tissue scaffolds using alginate and hepatocytes as bioink, either inkjet printed [25] or extrusion bioprinted [178,179,180], demonstrated upregulation of hepatic markers and excellent cell morphology. On the other hand, by adding ECM into alginate and gelatin bioinks, a printed 3D liver model containing human HepaRG liver cells not only maintained cell viability and metabolic functions, but also supported efficient adenoviral replication, showing its paradigmatic potential to be used for studies including viral vectors and infectious viruses [181].

##### Neural Tissue

Recent advances in 3D bioprinting make it possible to generate functional neural tissue. Schwann cells encapsulated in alginate bioink-based 3D bioprinted scaffolds enhanced expression of nerve growth factor [182], and promoted the cell alignment with uni-directional cues to guide the extension of dorsal root ganglion neurites along the printed strands [183], demonstrating the potential for nerve tissue engineering. In addition, Gu and colleagues used one bioink comprising alginate, carboxymethyl-chitosan, and agarose to fabricate 3D scaffolds encapsulating human neural stem cells, showing stem cell expansion and differentiation as the results of bicuculline-induced increased expression of calcium-responsive gamma-aminobutyric acid, and provided one neural model for investigation of human neural development, function, and disease [95,184].

##### Ocular Tissue

The retinal is critical to the formation of visual image in eyes. Many eye diseases may be linked to retinal pigment epithelium degeneration. 3D bioprinting can precisely deliver cells and biomolecules to provide a breakthrough for retina tissue engineering. For example, a hybrid retina construct including a PCL ultrathin membrane, human retinal pigmented epithelial (ARPE-19) cell monolayer and human retinoblastoma (Y79) cell-laden alginate/pluronic bioink was bioprinted and Y79 cells in bioink showed promising morphology and proliferation [26]. The successfully bioprinted retina model showed potential for ocular tissue applications.

##### Skeletal Muscle Tissue

Although skeletal muscles can self-repair relatively small damages, significant tissue loss cannot be self-restored, leading to severe trauma or invasive surgeries. Artificial skeletal muscle tissue has received increasing attention in the field of tissue engineering. Muscle precursor cell (C2C12 cell) laden PEG-fibrinogen and alginate bioink was used to print hydrogel fiber scaffolds by coaxial extrusion printing and demonstrated an enhanced myogenic differentiation with the formation of parallel aligned long-range myotubes like natural skeletal muscle [185]. Besides, compared to conventional cell-seeded scaffolds, C2C12 cell-laden pluronic/alginate blends-based 3D bioprinted tissue construct provided a cell density similar to that of native tissue, and also demonstrated cell alignment along the deposition direction to tailor the resulting cell histoarchitecture for native muscle tissues and showed high cell viability, as well as a significantly improved expression level of myogenic genes [186]. With the development of new biocompatible materials, carboxymethyl cellulose chemically functionalized with methacrylic anhydride, PEGDA blended into GelMA and methacrylic alginate have enabled the direct 3D printing of complex functional living tissues such as skeletal muscle fibers from C2C12 murine cells, and 3D bioprinting utilizing aforementioned biomaterials provided versatile, long lasting, and mechanically tunable scaffolds for myotube formation and alignment [187].

##### Skin Tissue and Sweat Gland

The skin, which can self-repair with minor injuries excluding extensive damage, is the largest organ in the human body and protects it from the external environment. Among the estimated 265,000 deaths per year caused by burns, many people lost skin function and had wound infection leading to multi-organ failure [188]. Engineered skin tissue constructs showed potential for artificial skin replacement [24,189]. Cell-laden alginate hydrogel blended with other biomaterials was added to a 3D bioprinter to fabricate bioactive skin scaffolds. For example, alginate, gelatin, and encapsulated hMSCs bioink-based skin tissue scaffold was 3D printed, showed complete attachment on the wound surface, and integrated with host tissues in a week. It showed biocompatibility for skin tissue engineering without toxicity and was not rejected [190]. Moreover, based on gelatin, alginate, fibrinogen and human dermal fibroblasts bioink, 3D printed highly organized full thickness skin (dermis + epidermis) presented all characteristics of human skin, at both molecular and macromolecular levels [191].

Body temperature is primarily controlled by sweating of the sweat glands in skin. However, severe skin burns may lead to failure of complete sweat gland regeneration with the hypertrophic scars and heat intolerance [192]. Alginate hydrogel-based 3D bioprinted hydrogel showed the ability to regenerate sweat glands. Adult epidermal progenitor cell-laden gelatin and alginate bioink was used to print 3D extracellular matrix scaffolds for a restrictive niche for epidermal progenitors to unilaterally differentiate into sweat gland cells. Animal implant results showed the functional restoration of sweat glands and the printed scaffold may have the potential for translational implications in regenerating sweat glands [193].

##### Vessel Systems

To maintain the functions of complex engineered tissue or organ with continuous nutrient requirement, the development of interconnected 3D vascular networks within the tissue or organ constructs is critical. 3D bioprinting technologies, in particular the coaxial printing technology [194,195] are promising for fabrication of highly organized 3D vascular networks. For example, alginate, GelMA, PEG-tetra-acrylate, HUVECs, and hMSCs bioink-based perfusable vascular structures with highly ordered arrangements were printed by a coaxial extrusion printer in a single-step process and supported the spread and proliferation of encapsulated endothelial and stem cells [36]. Besides, a small-sized artificial vessel was also fabricated based on alginate and hMSCs-derived endothelial-like cells bioink and showed upregulation of endothelial cell markers for vessel formation [196]. In addition to coaxial extrusion printing, inkjet printing printed alginate/fibrinogen droplets containing cells to fabricate circular patterns for microvessel constructs [197]. Hollow channels realized complex heterogeneous, hierarchical architectures and have strong potential for use in vascular tissue applications [198].

### 5.2. Further Applications

#### 5.2.1. Cell Therapy

A cell therapy is a medicinal product containing cells within materials or not, and is typically injected into a patient. However, one challenge, which is to maintain cell viability and function, lies in cell therapy application. The problem could be solved with 3D bioprinting by providing in-vivo-like microenvironment and diverse cell combination. For example, researchers successfully printed the hydrogel scaffolds containing alginate, gelatin, INS1E β-cells, human and mouse islets without affecting their morphology and viability by extrusion bioprinting. Further, pancreatic islets of multicellular constructs (islet in the inner layer and islet-related supporting cells in the outer layer) could also be constructed by co-axial bioprinting. After transplantation, blood vessels could grow into the pores of the construct [215] and the viability of pancreatic islets is well maintained after the 3D printing process [216]. They demonstrated the potential to produce functional Langerhans for some patients with type 1 diabetes. Moreover, through bioprinting technology, pancreatic islets encapsulated in macroporous 3D alginate and methylcellulose hydrogel constructs of defined geometry could continuously produce insulin and glucagon and still react to glucose stimulation albeit to a lesser degree than control islets [217].

Stem cell therapy has become a hot field in medical application. 3D bioprinting of stem cells directly into scaffolds offers great potential for the development of regenerative therapies [218]. Gu and colleagues printed a human iPSCs-containing scaffold in alginate, carboxymethyl-chitosan, and agarose hydrogel for in situ expansion and sequential multilineage differentiation [35]. Based on alginate hydrogel mixed with human neural stem cells, a layered lattice structure was created by 3D bioprinting and showed a viable and economical platform for human neural stem cells expansion that could be translated to clinical application [219]. However, it is important for stem cell 3D bioprinting to control shear stress in the printing process, because shear stress had effects on printing resolution and stem cell integrity [15]. The shear-thinning property of hydrogels can shield cells from high shear forces in bioprinting, which is mainly used for cell encapsulation and delivery. Thakur and colleagues used 2D nano-silcate reinforced k-carrageenan hydrogels for cell delivery. The shear-thinning characteristics of nanocomposite hydrogels are investigated for hMSCs delivery. The hMSCs showed high cell viability after injection and encapsulated cells showed a circular morphology [82].

#### 5.2.2. Drug Research System

Drug screening research evolved from traditional 2D cells or animal tests into patient-specific precision medicine in recent years because of the development of 3D cell cultures that use patient-derived cells and better mimic of the in vivo physiology [220]. In vitro physiological drug models based on 3D bioprinting technology have attracted increasing attention in pharmaceutical drug and toxicology screening for drug discovery and development (Table 4). Moreover, the assessment of efficacy and safety profiles of physiological models fabricated by 3D bioprinting has also been reviewed and some research confirmed that the efficacy and toxicity of drugs in 3D models are similar to that of the clinical data [221].

##### Marine-Derived Biomaterial based Bioprinting for Liver Drug Metabolism Model

Among in-vitro drug screening models, the liver organ is the focus of study due to its primary role in drug metabolism [222]. Fortunately, the convergence of 3D bioprinting technologies along with microfabrication and cell culture techniques enables the construction of a biomimetic 3D micro-tissue or organ model to serve as an in vitro platform for cell culture, and drug screening to provide further insights in cell–cell and cell–matrix interactions. Combining the development and application of natural marine biomaterials, a 3D microscale liver tissue analog was established by direct cell writing extrusion bioprinting using alginate hydrogel. Researchers observed collective drug metabolic function and suitability of liver tissue analog as a drug metabolism model and could assess drug pharmacokinetic profiles in planetary environments [208]. This biofabrication strategy of various in vitro 3D tissue analogs with specific tissue cells and complex microarchitecture could benefit clinical drug screening, by increasing the test efficacy and effects of the agent of interest.

##### Brain Tumor Model

**Figure 10:**
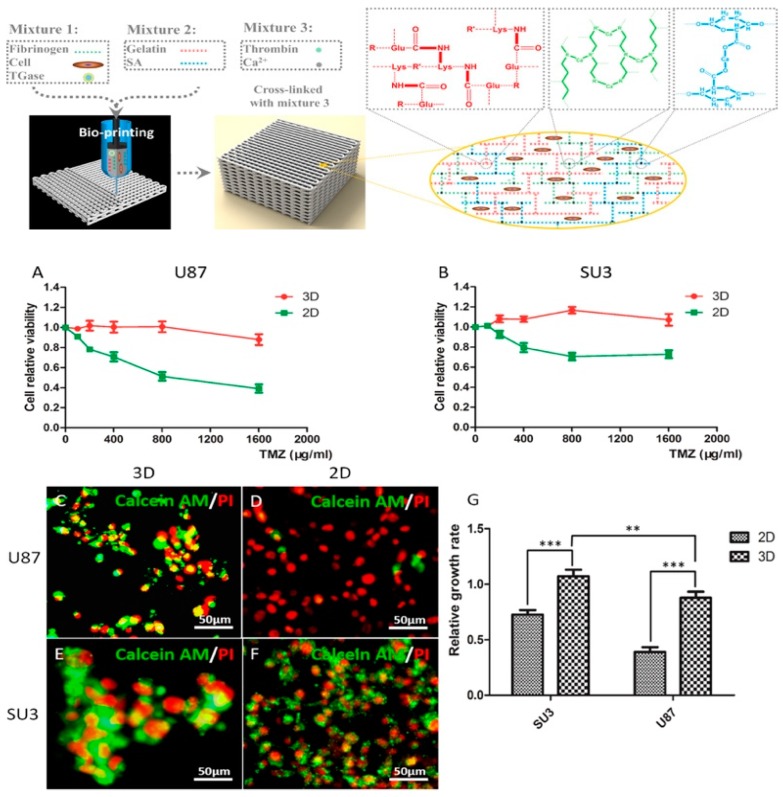
3D glioma tumor model by extrusion printing and application of susceptibility of tumor model to high drug concentration (**A**,**B**: TMZ-susceptibility of glioma cell lines at 3D and 2D. **C**–**F**: Live/dead images of TMZ treated glioma cells in 3D and 2D. G: relative growth rate of TMZ treated glioma cells in 3D and 2D) with permission from reference [225], Copyright IOPScience, 2019.

Despite recent advances in the treatment of brain tumors, the prediction of clinical efficacy of therapeutic drugs of patients with brain cancers, such as glioma, and neuroblastoma, is still challenging [223]. In-vitro glioma tumor model fabricated by 3D bioprinting technology with marine alginate [27,34] or chitosan [224] biomaterials and its drug pharmacokinetic study have made much progress in our lab. Dai and colleagues printed a brain glioma model by extrusion printing with alginate/gelatin/fibrinogen containing glioma stem cells. They showed drug susceptibility of the in vitro brain tumor models to temozolomide (TMZ), and the results demonstrated that cells in 3D printed tumor model were more resistant to TMZ than those in 2D monolayer model (Figure 10) [225]. In addition, for mimicking the glioma microenvironment with heterogeneous cell interaction, our group constructed shell-alginate and glioma stem cell GSC23/core-glioma cell line U118 (G/U) hydrogel microfibers with high cell viability by coaxial extrusion bioprinting. The results showed matrix metalloproteinase (MMP)-2, MMP9, vascular endothelial growth factor receptor-2 and O6-methylguanine-DNA methyltransferase, which are related to tumor invasion and drug resistance were significantly enhanced in G/U hydrogel microfibers, and that U118 cells derived from G/U microfibers had greater drug resistance to TMZ in vitro compared to U118 culture alone [226]. Besides, the neuroblastoma tumor model was also printed based on chitosan and gelatin hydrogel using an extruder and exhibited excellent viability for about one week [227].

##### Body Organ Tumor Model

The development of tumor study and drug therapy is limited by the inadequate understanding of tumorigenesis [228]. Due to ethical and safety limitations for tumor drug tests in clinical trials, 3D bioprinting technology has received increasing attention for preclinical tumor models with biomimetic physiological environments for in-vitro tumorgenesis study and anti-cancer drug screening [229,230]. For example, in vitro cervical tumor models based on marine-derived alginate, gelatin, fibrinogen and Hela cell bioinks were constructed by extrusion bioprinting, showing that cells in this model presented over 90% viability, and higher MMP protein expression and chemoresistance than those in 2D culture for cancer study [231]. Besides, 3D breast epithelial spheroids in alginate-based bioinks were used to bioprint breast tumor models and demonstrated they were more resistant to paclitaxel than individually printed breast cells, presenting the ability of 3D cellular structure bioprinting to capture the in-vivo microenvironment of tumor cells for tumor drug study [232]. Particularly, based on alginate and gelatin hydrogel, a bioprinted pituitary adenoma model demonstrated excellent proliferation and invasion of growth-hormone-secreting pituitary adenoma cells in our lab [233].

In the future, with the development of 3D bioprinting drug models, more model data could be further obtained and validated by using existing human data and in-vitro physiological drug models for precision medicine, and it will be a new form of therapeutics for treating many different diseases.

## 6. Conclusion and Outlook

To study in-vivo tissue-like 3D cell culture for regenerative medicine, 3D bioprinting has been paid increasing attention over the past decade [235,236]. However, one main challenge in the 3D bioprinting field has been to find biomaterials that are not only compatible with biological materials and the printing process, but also can provide the desired mechanical and functional properties for tissue constructs. Among all the hydrogels including synthetic and naturally sourced hydrogels, marine-derived hydrogels showed many advantages over synthetic and other sourced hydrogels as biopolymers from marine resources provide biological cues, low immune response, and excellent biocompatibility for tissue engineering applications compared with synthetic hydrogels [236]. They are abundant and not limited by religious restrictions, which are low in cost when compared with other sources [237,238]. However, it remains a challenge for applications of marine-derived biomaterials such as HA, collagen, and gelatin to control the consistent quality among different batches of products. In future, further improvement of isolation processes and industrial control technologies are needed to make marine natural biomaterials become a major choice for 3D bioprinting.

On the other hand, marine-derived biomaterials for 3D bioprinting applications are not without limitations in certain physical properties, such as mechanical strength, degradation, swelling ratio, and stiffness. Hydrogels formed via ionic, thermal, and photo triggers display relatively poor mechanical properties and eventually fail to maintain the desired 3D microgeometry integrity [235,239]. Therefore, the physical or chemical modifications of natural biomaterials could help achieve the potential of tissue-specific material biomimicry, such as methacrylate-conjugated natural polymers for application in tissue engineering [221]. In particular, a polysaccharidic chain can be modified to combine with functional groups for scaffold applications [115,227]. Alginate extracted from marine resource showed an influence on cell morphology and related gene expression in a structural or calcium-dependent manner [150], but alginate itself does not provide any cell attachment sites. Therefore, to increase cell interaction, alginate could be modified to link with bioactive peptides such as the RGD sequence and to show high cell adherence [70]. Also, alginate hydrogel induces cell attachment by combining with other biopolymers (gelatin, collagen, HA, chitosan, etc.) [71,95,149]. The strategies of chemically modified derivatives make these polysaccharides versatile biomaterials [240]. However, these strategies are limited as they could not achieve both the printability and mechanical properties needed for 3D microgeometry integrity and needed cellular function such as viability and differentiation at the same time, so it is one huge challenge for material scientists to find the ways to fulfill this need in the future.

Marine-derived biomaterials have emerged as commonly-used hydrogels in 3D bioprinting for regenerative medicine, such as tissue repair and regeneration during recent years [241,242]. Due to its ability to fabricate patient-specific 3D scaffolds containing various cells with well-controlled porous architecture, 3D bioprinting has become widely utilized for regenerative medicine. The ultimate aim of 3D bioprinting is to realize regeneration of artificial tissues or organs for complete replacements of diseased or damaged organs in patients. However, further work is still needed to create biomaterials that can satisfy all the requirements of artificial implanted tissue or organ regeneration still needing further work. For example, controlled degradation of biomaterials in vivo is difficult.

It is well known that the sea is a huge reservoir of natural biopolymers and life origin. Thus, raw marine materials will be a new resource for biomedical applications, especially for 3D bioprinting. Nevertheless, in-vivo testing is still needed to create combinations with other materials to improve performances of marine-derived materials. Marine-derived biomaterial-based regenerative medicine within a real clinical application still remains limited at this stage.

## Figures and Tables

**Table 1:** Comparison of 3D bioprinting technologies.

Bioprinting Technology	Inkjet	Extrusion	Stereolithography
Resolution	High (tens of micrometers)	Moderate (micrometers-millimeters)	High (micrometers)
Print speed	Fast	Slow	Fast
Cost	Low	Medium	Low
Bioink viscosity	Low	High	No limitation
Bioink gelation	Chemical, photocrosslinking	Chemical, enzymatic, thermal, photocrosslinking	Photocrosslinking
Cell density	Low	High	High
Representative marine-derived biomaterials for bioinks	Alginate, collagen	Alginate, carrageenan, chitosan, GelMA, collagen	GelMA, MeHA

**Table 2:** Summary of marine-derived biomaterials for 3D bioprinting.

Biomaterials	Marine Sources [56]	Category	Gelation Mechanism	Charge	Biodegardable	Cell Attachment	Cell Viability (%) [59]	Limitation	3D Bioprinting Methods	Ref.
Alginate	Brown algae	Natural	Ionic	anionic	Yes	Modified RGD	77-100	low biodegradability, lack cell binding domains	Inkjet printing, extrusion printing	[60,61]
Carrageenan	Red algae	Natural	Ionic and thermal	anionic	No	Yes	>80	Poor solubility, low biodegradability	Extrusion printing, stereolithographic printing	[58]
Chitosan	Arthropods, arthropods, marine algae	Natural	pH-sensitive	cationic	Yes	Yes	~75	Low mechanical integrity, poor solubility	Extrusion printing	[62]
HA	Fish tissue	Natural	Photo-sensitive MeHA	cationic	Yes	Yes	64.4	Low stability, no direct gelation	Extrusion printing, stereolithographic printing	[63,64]
Collagen	Fish tissue, gellyfish, marine sponges	Natural	Thermal	-	Yes	Yes	46-99	Low viscosity and mechanical integrity	Extrusion printing	[65]
Gelatin	Derivative of collagen	Natural	Thermal and Photo-sensitive GelMA	cationic	Yes	Yes	70-99.7	Low viscosity and mechanical integrity	Extrusion printing, inkjet printing, stereolithographic printing	[66]

**Table 3:** Marine-derived biomaterial hydrogels in 3D bioprinting for tissue engineering applications. hADMSCs, human adipose-derived mesenchymal stem cells; SMC, aortic root sinus smooth muscle cells; VIC, aortic valve leaflet interstitial cells; hAFS cells, human amniotic fluid–derived stem cells; SaOS-2 cells, human osteogenic sarcoma cells; RGD, Arginine-Glycine-Aspartic acid; GelMA, gelatin methacryloyl; hMSCs, human mesenchymal stem cells; HUVECs, human umbilical vein endothelial cells; iPSC-CMs, induced pluripotent stem cell-derived cardiomyocytes; CPCs, cartilage progenitor cells; C2C12 cell, muscle precursor cells.

Marine-Derived Biomaterial	Marine Biomaterial Resources	Application	Bioink Composites	3D Bioprinting Technology	Ref.
Alginate	Brown algae	Adipose tissue	Alginate/blood plasma/hADMSCs	Laser-assisted thermal inkjet printing	[146]
		Aortic valve	Alginate/gelatin/SMC/VIC	Extrusion bioprinting	[71]
			GelMA/polyethylene glycol diacrylate 3350/alginate/VIC/SMC	Extrusion bioprinting	[148]
		Bone tissue	Alginate/collagen/hAFS cells	Inkjet printing	[23]
			Alginate/multipotent stromal cells	Extrusion bioprinting	[149]
			Alginate/gelatin/SaOS-2 cells	Extrusion bioprinting	[199]
			Alginate/silica/biosilica/SaOS-2 cells	Extrusion bioprinting	[150]
			RGD-γ Alginate/polyethylene glycol methacryloyl/GelMA/hMSCs	Extrusion bioprinting	[158]
			Alginate/polylactic acid nanofibers/hADMSCs	Extrusion bioprinting	[151]
			Alginate/methylcellulose/nanosilicate clay/hMSCs	Extrusion bioprinting	[152]
			Alginate/polyvinyl alcohol/hydroxyapatite/MC3T3 mouse preosteoblasts	Extrusion bioprinting	[153,154]
			RGD-γ alginate/nano-hydroxyapatite/plasmid DNA/hMSCs	Extrusion bioprinting	[155]
			Collagen/alginate/fibrin/hMSCs	Jet bioprinting	[157]
			wood-based cellulose nanofibrils/bioactive glass/gelatin/alginate/Saos-2 cells/hMSCs	Extrusion bioprinting	[156]
		Cardiac tissue	Alginate/GelMA/endothelial cells	Extrusion bioprinting	[160]
			Alginate/human coronary artery endothelial cells	Extrusion bioprinting	[163]
			Carbon nanotubes/methacrylated collagen/alginate/human coronary artery endothelial cells	Extrusion bioprinting	[161]
			Alginate/PEG-fibrinogen/HUVECs/iPSC-CMs	Coaxial extrusion bioprinting	[162]
		Cartilage tissue	Alginate/osteoblasts/chondrocytes	Extrusion bioprinting	[165]
			Alginate/CPCs	Coaxial extrusion bioprinting	[166]
			Gellan/alginate/BioCartilage/chondrocytes	Coaxial extrusion bioprinting	[200]
			Alginate/chondrocyte	Extrusion bioprinting	[167,168,169]
			Cellulose/alginate/chondrocyte	Extrusion bioprinting	[72,171,172,173]
			Alginate/GelMA/chondroitin sulfate amino ethyl methacrylate/methacrylated hyaluronic acid/hMSCs	Coaxial extrusion bioprinting	[201]
			Alginate/agarose/GelMA/BioINK™/hMSCs	Extrusion bioprinting	[202]
			Cellulose/alginate/chondrocytes/hMSCs	Extrusion bioprinting	[203,204]
			Alginate/polylactic acid/chondrocyte	Extrusion bioprinting	[205]
			Cellulose/alginate/iPSCs	Extrusion bioprinting	[206]
			Cellulose/alginate/chondrocyte	Inkjet printing	[24]
			Collagen/alginate/chondrocyte	Extrusion bioprinting	[207]
			Hydroxyapatite/alginate/chondrocyte	Extrusion bioprinting	[174]
			Cartilage decellularized extracellular matrix/alginate/hMSCs	Extrusion bioprinting	[175]
		Dental tissue	Alginate/ECM/mouse odontoblast-like OD21 cells/human dental stem cells from the apical papilla	Extrusion bioprinting	[176]
			Alginate/gelatin/human Dental Pulp Stem Cells	Extrusion bioprinting	[177]
		Liver tissue	Alginate/hepatocyte-like cells	Inkjet printing	[25]
			Alginate/HepG2 cells	Extrusion bioprinting	[178,208]
			Alginate/mouse primary hepatocytes	Extrusion bioprinting	[179]
			Alginate/gelatin/ECM/human HepaRG liver cells	Extrusion bioprinting	[181]
			Alginate/mouse-induced hepatocyte-like cells	Extrusion bioprinting	[180]
			Alginate/cellulose nanocrystals/fibroblast/hepatoma cells	Extrusion bioprinting	[209]
		Neural tissue	Alginate/carboxymethyl-chitosan/agarose/human neural stem cells	Extrusion bioprinting	[95,184]
			Alginate/gelatin/Schwann cell RSC96s	Coaxial extrusion bioprinting	[182]
			Alginate/fibrin/HA/RGD peptide/Schwann cell	Extrusion bioprinting	[183]
		Ocular tissue	Alginate/pluronic/Y79 human retinoblastoma cell	Inkjet printing	[26]
		Skeletal muscle tissue	PEG-Fibrinogen/alginate/C2C12 cell	Coaxial extrusion bioprinting	[185]
			Pluronic/alginate/C2C12 cell	Extrusion bioprinting	[186]
			GelMA/PEG-diacrylate/carboxymethyl cellulose chemically functionalized with methacrylic anhydride/methacryloyl Alginate/C2C12 cell	Extrusion bioprinting	[187]
		Skin tissue	Gelatin/alginate/hMSCs	Extrusion bioprinting	[190]
			Gelatin/alginate/fibrinogen/human dermal fibroblasts	Extrusion bioprinting	[191]
			Gelatin/alginate/human skin primary fibroblast cells	Extrusion bioprinting	[189]
			Cellulose/alginate/primary human dermal fibroblasts/human nasal chondrocytes	Extrusion bioprinting	[24]
		Sweat gland	Gelatin/alginate/epidermal progenitor cells	Extrusion bioprinting	[193]
		Vessel system	Alginate/CPCs	Coaxial extrusion bioprinting	[166,195]
			Alginate/L929 mouse fibroblasts	Coaxial extrusion bioprinting	[194]
			Alginate/GelMA/polyethylene glycol tetra-acrylate/HUVECs/hMSCs	Coaxial extrusion bioprinting	[36]
			Alginate/fibroblasts/smooth muscle cells	Coaxial extrusion bioprinting	[210]
			Alginate/fibrinogen/endothelial cells	Inkjet printing	[197]
			Alginate/endothelial cells/fibroblasts	Coaxial extrusion bioprinting	[198]
			Alginate/endothelial-like cells	Coaxial extrusion bioprinting	[196]
			Alginate/GelMA/PEG/human urothelial cells/human bladder smooth muscle cells/human umbilical vein endothelial cells/human smooth muscle cells	Coaxial extrusion bioprinting	[211]
Carrageenan	Red algae	Cartilage tissue	κ-carrageenan/hADMSCs/human nasal chondrocytes	Model pattern	[170]
			MA-κ-carrageenan/2D nanosilicates/hMSC	Extrusion bioprinting	[82]
		Tissue scaffolds	MA-κ-carrageenan/NIH-3T3 fibroblast cells/MC3T3 mouse preosteoblasts/hMSCs	Model pattern	[81]
			κ-carrageenan/2D nanosilicates/MC3T3 mouse preosteoblasts	Extrusion bioprinting	[212]
			GelMA/κ-carrageenan/nanosilicates/hMSCs	Extrusion bioprinting	[213]
			Carrageenan/alginate/hADMSCs	Extrusion bioprinting	[91]
Chitosan	Shell	Bone tissue	Chitosan/hydroxyapatite/MC3T3 mouse preosteoblasts	Extrusion bioprinting	[102]
		Cartilage tissue	Hyaluronate/chitosan/adipic acid dihydrazide/ATDC5 chondrocyte	Extrusion bioprinting	[164]
			Chitosan/oxidized chondroitin sulfate/hADMSCs	Extrusion bioprinting	[143]
		Neural Tissues	Alginate/carboxymethyl-chitosan/agarose/human neural stem cells	Extrusion bioprinting	[95,184]
		Tissue scaffolds	Gelatin/alginate/carboxymethyl chitosan/hMSCs	Extrusion bioprinting	[214]
Gelatin	Fish tissue	Tissue scaffolds	Alginate/fish GelMA/NIH-3T3 fibroblast cells	Coaxial extrusion bioprinting	[8]

**Table 4:** Resume of marine-derived biomaterial hydrogels in 3D bioprinting for tumor model.

Marine-Derived Biomaterial	Marine Biomaterial Resources	Tumor Model	Bioink Composites	3D Bioprinting Technology	Ref.
Alginate	Brown algae	Cervical	Gelatin/alginate/fibrinogen/Hela cells	Extrusion bioprinting	[231]
			Alginate/U87 glioma cell line	Extrusion bioprinting	[34]
		Glioma	Gelatin/alginate/fibrinogen/glioma stem cell	Extrusion bioprinting	[27,225]
			Gelatin/alginate/fibrinogen/glioma stem cell/human mesenchymal stem cells	Coaxial extrusion bioprinting	[234]
			Alginate/glioma stem cell/U118 glioma cell line	Coaxial extrusion bioprinting	[226]
		Breast	Alginate/gelatin/MDA-MB-231 breast cancer cells	Extrusion bioprinting	[230]
			Alginate/gelatin or collagen/breast epithelial cells	Extrusion bioprinting	[232]
		Lung	Alginate/gelatin/lung cancer cell A549/95-D	Extrusion bioprinting	[229]
		Pituitary adenoma	Alginate/gelatin/rat pituitary adenoma GH3 cells	Extrusion bioprinting	[233]
Chitosan	Shell	Glioma	Chitosan/HA/glioma stem cell	Extrusion bioprinting	[224]
		Neuroblastoma	Chitosan/gelatin/neuroblastoma cells	Extrusion bioprinting	[227]

## References

[1] Kang H.W., Lee S.J., Ko I.K., Kengla C., Yoo J.J., Atala A. (2016). A 3D bioprinting system to produce human-scale tissue constructs with structural integrity. Nat. Biotechnol..

[2] Murphy S.V., Atala A. (2014). 3D bioprinting of tissues and organs. Nat. Biotechnol..

[3] Skardal A., Atala A., Yoo J.J. (2015). Bioprinting Essentials of Cell and Protein Viability. Essentials of 3D Biofabrication and Translation.

[4] Chia H.N., Wu B.M. (2015). Recent advances in 3D printing of biomaterials. J. Biol. Eng..

[5] Skardal A., Devarasetty M., Kang H.W., Mead I., Bishop C., Shupe T., Lee S.J., Jackson J., Yoo J., Soker S. (2015). A hydrogel bioink toolkit for mimicking native tissue biochemical and mechanical properties in bioprinted tissue constructs. Acta Biomater..

[6] D’Ayala G.G., Malinconico M., Laurienzo P. (2008). Marine Derived Polysaccharides for Biomedical Applications: Chemical Modification Approaches. Molecules.

[7] Luz G.M., Mano J.F. (2010). Mineralized structures in nature: Examples and inspirations for the design of new composite materials and biomaterials. Compos. Sci. Technol..

[8] Zhang X., Kim G.J., Kang M.G., Lee J.K., Seo J.W., Do J.T., Hong K., Cha J.M., Shin S.R., Bae H. (2018). Marine Biomaterial-Based Bioinks for Generating 3D Printed Tissue Constructs. Mar. Drugs.

[9] Park T.Y., Yang Y.J., Ha D.H., Cho D.W., Cha H.J. (2019). Marine-derived natural polymer-based bioprinting ink for biocompatible, durable, and controllable 3D constructs. Biofabrication.

[10] Ozbolat I.T., Hospodiuk M. (2016). Current advances and future perspectives in extrusion-based bioprinting. Biomaterials.

[11] Hunt N.C., Grover L.M. (2010). Cell encapsulation using biopolymer gels for regenerative medicine. Biotechnol. Lett..

[12] Li Z., Kawashita M. (2011). Current progress in inorganic artificial biomaterials. J. Artif. Organs.

[13] Panwar A., Tan L.P. (2016). Current Status of Bioinks for Micro-Extrusion-Based 3D Bioprinting. Molecules.

[14] Malda J., Visser J., Melchels F.P., Jüngst T., Hennink W.E., Dhert W.J.A., Groll J., Hutmacher D.W. (2013). 25th Anniversary Article: Engineering Hydrogels for Biofabrication. Adv. Mater..

[15] Blaeser A., Duarte Campos D.F., Puster U., Richtering W., Stevens M.M., Fischer H. (2016). Controlling Shear Stress in 3D Bioprinting is a Key Factor to Balance Printing Resolution and Stem Cell Integrity. Adv. Healthc. Mater..

[16] Li H., Tan Y.J., Leong K.F., Li L. (2017). 3D Bioprinting of Highly Thixotropic Alginate/Methylcellulose Hydrogel with Strong Interface Bonding. ACS Appl. Mater. Interfaces.

[17] Tan Y.J., Leong K.F., An J., Chian K.S., Tan X., Yeong W.Y. (2015). Fabrication and in vitro analysis of tubular scaffolds by melt-drawing for esophageal tissue engineering. Mater. Lett..

[18] Carrow J.K., Kerativitayanan P., Jaiswal M.K., Lokhande G., Gaharwar A.K., Atala A., Yoo J.J. (2015). Polymers for Bioprinting. Essentials of 3D Biofabrication and Translation.

[19] Gudapati H., Dey M., Ozbolat I. (2016). A comprehensive review on droplet-based bioprinting: Past, present and future. Biomaterials.

[20] Park J.A., Yoon S., Kwon J., Now H., Kim Y.K., Kim W.J., Yoo J.Y., Jung S. (2017). Freeform micropatterning of living cells into cell culture medium using direct inkjet printing. Sci. Rep..

[21] Miller E.D., Li K., Kanade T., Weiss L.E., Walker L.M., Campbell P.G. (2011). Spatially directed guidance of stem cell population migration by immobilized patterns of growth factors. Biomaterials.

[22] Xu T., Rohozinski J., Zhao W., Moorefield E.C., Atala A., Yoo J.J. (2009). Inkjet-Mediated Gene Transfection into Living Cells Combined with Targeted Delivery. Tissue Eng. Part A.

[23] De Coppi P., Bartsch G., Siddiqui M.M., Xu T., Santos C.C., Perin L., Mostoslavsky G., Serre A.C., Snyder E.Y., Yoo J.J. (2007). Isolation of amniotic stem cell lines with potential for therapy. Nat. Biotechnol..

[24] Thayer P.S., Orrhult L.S., Martinez H. (2018). Bioprinting of Cartilage and Skin Tissue Analogs Utilizing a Novel Passive Mixing Unit Technique for Bioink Precellularization. J. Vis. Exp..

[25] Faulkner-Jones A., Fyfe C., Cornelissen D.J., Gardner J., King J., Courtney A., Shu W. (2015). Bioprinting of human pluripotent stem cells and their directed differentiation into hepatocyte-like cells for the generation of mini-livers in 3D. Biofabrication.

[26] Shi P., Edgar T.Y.S., Yeong W.Y., Laude A. (2017). Hybrid three-dimensional (3D) bioprinting of retina equivalent for ocular research. Int. J. Bioprint..

[27] Wang X., Li X., Dai X., Zhang X., Zhang J., Xu T., Lan Q. (2018). Bioprinting of glioma stem cells improves their endotheliogenic potential. Colloids Surf. B Biointerfaces.

[28] Doraiswamy A., Dunaway T.M., Wilker J.J., Narayan R.J. (2009). Inkjet printing of bioadhesives. J. Biomed. Mater. Res. Part B Appl. Biomater..

[29] Cui X., Boland T. (2009). Human microvasculature fabrication using thermal inkjet printing technology. Biomaterials.

[30] Ru C., Luo J., Xie S., Sun Y. (2014). A review of non-contact micro- and nano-printing technologies. J. Micromech. Microeng..

[31] Yan Y., Wang X., Pan Y., Liu H., Cheng J., Xiong Z., Lin F., Wu R., Zhang R., Lu Q. (2005). Fabrication of viable tissue-engineered constructs with 3D cell-assembly technique. Biomaterials.

[32] Elloumi-Hannachi I., Yamato M., Okano T. (2010). Cell sheet engineering: A unique nanotechnology for scaffold-free tissue reconstruction with clinical applications in regenerative medicine. J. Intern. Med..

[33] Tabriz A.G., Hermida M.A., Leslie N.R., Shu W. (2015). Three-dimensional bioprinting of complex cell laden alginate hydrogel structures. Biofabrication.

[34] Gu Q., Tomaskovic-Crook E., Wallace G.G., Crook J.M. (2017). 3D Bioprinting Human Induced Pluripotent Stem Cell Constructs for In Situ Cell Proliferation and Successive Multilineage Differentiation. Adv. Healthc. Mater..

[35] Jia W., Gungor-Ozkerim P.S., Zhang Y.S., Yue K., Zhu K., Liu W., Pi Q., Byambaa B., Dokmeci M.R., Shin S.R. (2016). Direct 3D bioprinting of perfusable vascular constructs using a blend bioink. Biomaterials.

[36] Wu Z., Su X., Xu Y., Kong B., Sun W., Mi S. (2016). Bioprinting three-dimensional cell-laden tissue constructs with controllable degradation. Sci. Rep..

[37] Wang L.L., Highley C.B., Yeh Y.C., Galarraga J.H., Uman S., Burdick J.A. (2018). Three-dimensional extrusion bioprinting of single- and double-network hydrogels containing dynamic covalent crosslinks. J. Biomed. Mater. Res. Part A.

[38] Duarte Campos D.F., Blaeser A., Korsten A., Neuss S., Jäkel J., Vogt M., Fischer H. (2015). The Stiffness and Structure of Three-Dimensional Printed Hydrogels Direct the Differentiation of Mesenchymal Stromal Cells Toward Adipogenic and Osteogenic Lineages. Tissue Eng. Part A.

[39] Cohen D.L., Lipton J.I., Bonassar L.J., Lipson H. (2010). Additive manufacturing forin siturepair of osteochondral defects. Biofabrication.

[40] Ouyang L., Yao R., Chen X., Na J., Sun W. (2015). 3D printing of HEK 293FT cell-laden hydrogel into macroporous constructs with high cell viability and normal biological functions. Biofabrication.

[41] Hao T., Wen N., Cao J.K., Wang H.B., Lü S.H., Liu T., Lin Q.X., Duan C.M., Wang C.Y. (2010). The support of matrix accumulation and the promotion of sheep articular cartilage defects repair in vivo by chitosan hydrogels. Osteoarthr. Cartil..

[42] Möller S., Weisser J., Bischoff S., Schnabelrauch M. (2007). Dextran and hyaluronan methacrylate based hydrogels as matrices for soft tissue reconstruction. Biomol. Eng..

[43] Gerecht S., Burdick J.A., Ferreira L.S., Townsend S.A., Langer R., Vunjak-Novakovic G. (2007). Hyaluronic acid hydrogel for controlled self-renewal and differentiation of human embryonic stem cells. Proc. Natl. Acad. Sci. USA.

[44] Ouyang L., Highley C.B., Sun W., Burdick J.A. (2017). A Generalizable Strategy for the 3D Bioprinting of Hydrogels from Nonviscous Photo-crosslinkable Inks. Adv. Mater..

[45] Onoe H., Okitsu T., Itou A., Kato-Negishi M., Gojo R., Kiriya D., Sato K., Miura S., Iwanaga S., Kuribayashi-Shigetomi K. (2013). Metre-long cell-laden microfibres exhibit tissue morphologies and functions. Nat. Mater..

[46] Sugai K., Nishimura S., Kato-Negishi M., Onoe H., Iwanaga S., Toyama Y., Matsumoto M., Takeuchi S., Okano H., Nakamura M. (2015). Neural stem/progenitor cell-laden microfibers promote transplant survival in a mouse transected spinal cord injury model. J. Neurosci. Res..

[47] Ouyang L., Yao R., Mao S., Chen X., Na J., Sun W. (2015). Three-dimensional bioprinting of embryonic stem cells directs highly uniform embryoid body formation. Biofabrication.

[48] Ouyang L., Yao R., Zhao Y., Sun W. (2016). Effect of bioink properties on printability and cell viability for 3D bioplotting of embryonic stem cells. Biofabrication.

[49] D’Urso P.S., Effeney D.J., Earwaker W.J., Barker T.M., Redmond M.J., Thompson R.G., Tomlinson F.H. (2000). Custom cranioplasty using stereolithography and acrylic. Br. J. Plast. Surg..

[50] Cooke M.N., Fisher J.P., Dean D., Rimnac C., Mikos A.G. (2003). Use of stereolithography to manufacture critical-sized 3D biodegradable scaffolds for bone ingrowth. J. Biomed. Mater. Res. Part B.

[51] Lee K.W., Wang S., Fox B.C., Ritman E.L., Yaszemski M.J., Lu L. (2007). Poly(propylene fumarate) bone tissue engineering scaffold fabrication using stereolithography: Effects of resin formulations and laser parameters. Biomacromolecules.

[52] Zhang X., Jiang X.N., Sun C. (1999). Micro-stereolithography of polymeric and ceramic microstructures. Sens. Actuators A Phys..

[53] Billiet T., Gevaert E., De Schryver T., Cornelissen M., Dubruel P. (2014). The 3D printing of gelatin methacrylamide cell-laden tissue-engineered constructs with high cell viability. Biomaterials.

[54] Lam T., Dehne T., Krüger J.P., Hondke S., Endres M., Thomas A., Lauster R., Sittinger M., Kloke L. (2019). Photopolymerizable gelatin and hyaluronic acid for stereolithographic 3D bioprinting of tissue-engineered cartilage. J. Biomed. Mater. Res. Part B Appl. Biomater..

[55] Murado M.A., Montemayor M.I., Cabo M.L., Vázquez J.A., González M.P. (2012). Optimization of extraction and purification process of hyaluronic acid from fish eyeball. Food Bioprod. Process..

[56] Silva T.H., Alves A., Ferreira B.M., Oliveira J.M., Reys L.L., Ferreira R.J.F., Sousa R.A., Silva S.S., Mano J.F., Reis R.L. (2012). Materials of marine origin: A review on polymers and ceramics of biomedical interest. Int. Mater. Rev..

[57] McHugh D.J., Hernández-Carmona G., Luz Arvizu-Higuera D., Rodríguez-Montesinos Y.E. (2001). Pilot plant scale extraction of alginates from Macrocystis pyrifera 3. Precipitation, bleaching and conversion of calcium alginate to alginic acid. J. Appl. Phycol..

[58] Yegappan R., Selvaprithiviraj V., Amirthalingam S., Jayakumar R. (2018). Carrageenan based hydrogels for drug delivery, tissue engineering and wound healing. Carbohydr. Polym..

[59] Donderwinkel I., van Hest J.C.M., Cameron N.R. (2017). Bio-inks for 3D bioprinting: Recent advances and future prospects. Polym. Chem..

[60] Pillai C.K.S., Paul W., Sharma C.P. (2009). Chitin and chitosan polymers: Chemistry, solubility and fiber formation. Prog. Polym. Sci..

[61] Freeman F.E., Kelly D.J. (2017). Tuning Alginate Bioink Stiffness and Composition for Controlled Growth Factor Delivery and to Spatially Direct MSC Fate within Bioprinted Tissues. Sci. Rep..

[62] Ravi Kumar M.N.V. (2000). A review of chitin and chitosan applications. React. Funct. Polym..

[63] Liao Y.H., Jones S.A., Forbes B., Martin G.P., Brown M.B. (2005). Hyaluronan: Pharmaceutical characterization and drug delivery. Drug Deliv..

[64] Poldervaart M.T., Goversen B., de Ruijter M., Abbadessa A., Ferry P.W.M., Öner F.C., Dhert W.J.A., Vermonden T., Alblas J. (2017). 3D bioprinting of methacrylated hyaluronic acid (MeHA) hydrogel with intrinsic osteogenicity. PLoS ONE.

[65] Kim J.E., Kim S.H., Jung Y. (2016). Current status of three-dimensional printing inks for soft tissue regeneration. Tissue Eng. Regen. Med..

[66] Zhang L., Li G., Shi M., Liu H.H., Ge S., Ou Y., Flanagan J.G., Chen L. (2017). Establishment and Characterization of an Acute Model of Ocular Hypertension by Laser-Induced Occlusion of Episcleral Veins. Investig. Ophthalmol. Vis. Sci..

[67] Strand B.L., Morch Y.A., Skjak-Braek G. (2000). Alginate as immobilization matrix for cells. Minerva Biotecnol..

[68] Gomez C.G., Pérez Lambrecht M.V., Lozano J.E., Rinaudo M., Villar M.A. (2009). Influence of the extraction–purification conditions on final properties of alginates obtained from brown algae (Macrocystis pyrifera). Int. J. Biol. Macromol..

[69] Drury J.L., Dennis R.G., Mooney D.J. (2004). The tensile properties of alginate hydrogels. Biomaterials.

[70] Dalheim M.Ø., Vanacker J., Najmi M.A., Aachmann F.L., Strand B.L., Christensen B.E. (2016). Efficient functionalization of alginate biomaterials. Biomaterials.

[71] Duan B., Hockaday L.A., Kang K.H., Butcher J.T. (2013). 3D Bioprinting of heterogeneous aortic valve conduits with alginate/gelatin hydrogels. J. Biomed. Mater. Res. Part A.

[72] Markstedt K., Mantas A., Tournier I., Martínez Ávila H., Hägg D., Gatenholm P. (2015). 3D Bioprinting Human Chondrocytes with Nanocellulose–Alginate Bioink for Cartilage Tissue Engineering Applications. Biomacromolecules.

[73] Tønnesen H.H., Karlsen J. (2002). Alginate in Drug Delivery Systems. Drug Dev. Ind. Pharm..

[74] Klöck G., Pfeffermann A., Ryser C., Gröhn P., Kuttler B., Hahn H.J., Zimmermann U. (1997). Biocompatibility of mannuronic acid-rich alginates. Biomaterials.

[75] Lee K.Y., Mooney D.J. (2012). Alginate: Properties and biomedical applications. Prog. Polym. Sci..

[76] Rioux L.E., Turgeon S.L., Beaulieu M. (2007). Rheological characterisation of polysaccharides extracted from brown seaweeds. J. Sci. Food Agric..

[77] Kuo C.K., Ma P.X. (2001). Ionically crosslinked alginate hydrogels as scaffolds for tissue engineering: Part 1. Structure, gelation rate and mechanical properties. Biomaterials.

[78] Khalil S., Sun W. (2009). Bioprinting Endothelial Cells With Alginate for 3D Tissue Constructs. J. Biomech. Eng..

[79] Lee K.Y., Kong H.J., Larson R.G., Mooney D.J. (2003). Hydrogel Formation via Cell Crosslinking. Adv. Mater..

[80] Cunha L., Grenha A. (2016). Sulfated Seaweed Polysaccharides as Multifunctional Materials in Drug Delivery Applications. Mar. Drugs.

[81] Mihaila S.M., Gaharwar A.K., Reis R.L., Marques A.P., Gomes M.E., Khademhosseini A. (2013). Photocrosslinkable Kappa-Carrageenan Hydrogels for Tissue Engineering Applications. Adv. Healthc. Mater..

[82] Thakur A., Jaiswal M.K., Peak C.W., Carrow J.K., Gentry J., Dolatshahi-Pirouz A., Gaharwar A.K. (2016). Injectable shear-thinning nanoengineered hydrogels for stem cell delivery. Nanoscale.

[83] Guibet M., Colin S., Barbeyron T., Genicot S., Kloareg B., Michel G., Helbert W. (2007). Degradation of λ-carrageenan by Pseudoalteromonas carrageenovora λ-carrageenase: A new family of glycoside hydrolases unrelated to κ- and ι-carrageenases. Biochem. J..

[84] Li H., Tan Y.J., Liu S., Li L. (2018). Three-Dimensional Bioprinting of Oppositely Charged Hydrogels with Super Strong Interface Bonding. ACS Appl. Mater. Interfaces.

[85] Li H., Tan Y.J., Li L. (2018). A strategy for strong interface bonding by 3D bioprinting of oppositely charged κ-carrageenan and gelatin hydrogels. Carbohydr. Polym..

[86] Campo V.L., Kawano D.F., Silva D.B.d., Carvalho I. (2009). Carrageenans: Biological properties, chemical modifications and structural analysis—A review. Carbohydr. Polym..

[87] Yuan H., Zhang W., Li X., Lü X., Li N., Gao X., Song J. (2005). Preparation and in vitro antioxidant activity of κ-carrageenan oligosaccharides and their oversulfated, acetylated, and phosphorylated derivatives. Carbohydr. Res..

[88] Zhu M., Ge L., Lyu Y., Zi Y., Li X., Li D., Mu C. (2017). Preparation, characterization and antibacterial activity of oxidized κ-carrageenan. Carbohydr. Polym..

[89] Aparna V., Melge A.R., Rajan V.K., Biswas R., Jayakumar R., Gopi Mohan C. (2018). Carboxymethylated ɩ-carrageenan conjugated amphotericin B loaded gelatin nanoparticles for treating intracellular Candida glabrata infections. Int. J. Biol. Macromol..

[90] Popa E.G., Reis R.L., Gomes M.E. (2015). Seaweed polysaccharide-based hydrogels used for the regeneration of articular cartilage. Crit. Rev. Biotechnol..

[91] Kim M.H., Lee Y.W., Jung W.K., Oh J., Nam S.Y. (2019). Enhanced rheological behaviors of alginate hydrogels with carrageenan for extrusion-based bioprinting. J. Mech. Behav. Biomed. Mater..

[92] Peniche C., Arguelles-Monal W., Goycoolea F.M. (2008). Chitin and Chitosan: Major Sources, Properties and Applications. Monomers, Polymers and Composites from Renewable Resources.

[93] Lizardi-Mendoza J., Argüelles Monal W.M., Goycoolea Valencia F.M., Bautista-Baños S., Romanazzi G., Jiménez-Aparicio A. (2016). Chemical Characteristics and Functional Properties of Chitosan. Chitosan in the Preservation of Agricultural Commodities.

[94] Verlee A., Mincke S., Stevens C.V. (2017). Recent developments in antibacterial and antifungal chitosan and its derivatives. Carbohydr. Polym..

[95] Gu Q., Tomaskovic-Crook E., Lozano R., Chen Y., Kapsa R.M., Zhou Q., Wallace G.G., Crook J.M. (2016). Functional 3D Neural Mini-Tissues from Printed Gel-Based Bioink and Human Neural Stem Cells. Adv. Healthc. Mater..

[96] Chang K.L.B., Tsai G., Lee J., Fu W.R. (1997). Heterogeneous N-deacetylation of chitin in alkaline solution. Carbohydr. Res..

[97] Francis Suh J.K., Matthew H.W.T. (2000). Application of chitosan-based polysaccharide biomaterials in cartilage tissue engineering: A review. Biomaterials.

[98] Tuzlakoglu K., Alves C.M., Mano J.F., Reis R.L. (2004). Production and Characterization of Chitosan Fibers and 3-D Fiber Mesh Scaffolds for Tissue Engineering Applications. Macromol. Biosci..

[99] Thomas V., Yallapu M.M., Sreedhar B., Bajpai S.K. (2009). Fabrication, Characterization of Chitosan/Nanosilver Film and Its Potential Antibacterial Application. J. Biomater. Sci. Polym. Ed..

[100] Chung Y.C., Chen C.Y. (2008). Antibacterial characteristics and activity of acid-soluble chitosan. Bioresour. Technol..

[101] Goy R.C., Britto D.D., Assis O.B.G. (2009). A review of the antimicrobial activity of chitosan. Polímeros.

[102] Demirtaş T.T., Irmak G., Gümüşderelioğlu M. (2017). A bioprintable form of chitosan hydrogel for bone tissue engineering. Biofabrication.

[103] Hayes M., Carney B., Slater J., Brück W. (2008). Mining marine shellfish wastes for bioactive molecules: Chitin and chitosan ndash; Part A: Extraction methods. Biotechnol. J..

[104] Matet M., Heuzey M.C., Pollet E., Ajji A., Avérous L. (2013). Innovative thermoplastic chitosan obtained by thermo-mechanical mixing with polyol plasticizers. Carbohydr. Polym..

[105] Meyer K., Palmer J.W. (1934). The polysaccharide of the vitreous humor. J. Biol. Chem..

[106] Laurent T.C., Laurent U.B., Fraser J.R. (1995). Functions of hyaluronan. Ann. Rheum. Dis..

[107] Scott J.E., Heatley F. (2002). Biological Properties of Hyaluronan in Aqueous Solution Are Controlled and Sequestered by Reversible Tertiary Structures, Defined by NMR Spectroscopy. Biomacromolecules.

[108] Burdick J.A., Chung C., Jia X., Randolph M.A., Langer R. (2005). Controlled Degradation and Mechanical Behavior of Photopolymerized Hyaluronic Acid Networks. Biomacromolecules.

[109] Barbucci R., Lamponi S., Borzacchiello A., Ambrosio L., Fini M., Torricelli P., Giardino R. (2002). Hyaluronic acid hydrogel in the treatment of osteoarthritis. Biomaterials.

[110] Uthman I., Raynauld J.P., Haraoui B. (2003). Intra-articular therapy in osteoarthritis. Postgrad. Med. J..

[111] Medina J.M., Thomas A., Craig R.D. (2006). Knee osteoarthritis: Should your patient opt for hyaluronic acid injection?. J. Fam. Pract..

[112] Sakai S., Ohi H., Hotta T., Kamei H., Taya M. (2018). Differentiation potential of human adipose stem cells bioprinted with hyaluronic acid/gelatin-based bioink through microextrusion and visible light-initiated crosslinking. Biopolymers.

[113] Law N., Doney B., Glover H., Qin Y.H., Aman Z.M., Sercombe T.B., Liew L.J., Dilley R.J., Doyle B.J. (2018). Characterisation of hyaluronic acid methylcellulose hydrogels for 3D bioprinting. J. Mech. Behav. Biomed. Mater..

[114] Pescosolido L., Schuurman W., Malda J., Matricardi P., Alhaique F., Coviello T., van Weeren P.R., Dhert W.J.A., Hennink W.E., Vermonden T. (2011). Hyaluronic Acid and Dextran-Based Semi-IPN Hydrogels as Biomaterials for Bioprinting. Biomacromolecules.

[115] Tan H., Chu C.R., Payne K.A., Marra K.G. (2009). Injectable in situ forming biodegradable chitosan–hyaluronic acid based hydrogels for cartilage tissue engineering. Biomaterials.

[116] Abraham L.C., Zuena E., Perez-Ramirez B., Kaplan D.L. (2008). Guide to collagen characterization for biomaterial studies. J. Biomed. Mater. Res. Part B.

[117] Shoulders M.D., Raines R.T. (2009). Collagen Structure and Stability. Annu. Rev. Biochem..

[118] Duarte Campos D.F., Rohde M., Ross M., Anvari P., Blaeser A., Vogt M., Panfil C., Yam G.H.F., Mehta J.S., Fischer H. (2019). Corneal bioprinting utilizing collagen-based bioinks and primary human keratocytes. J. Biomed. Mater. Res. Part A.

[119] Parenteau-Bareil R., Gauvin R., Berthod F. (2010). Collagen-Based Biomaterials for Tissue Engineering Applications. Materials.

[120] Glowacki J., Mizuno S. (2008). Collagen scaffolds for tissue engineering. Biopolymers.

[121] Isaacson A., Swioklo S., Connon C.J. (2018). 3D bioprinting of a corneal stroma equivalent. Exp. Eye Res..

[122] Albanna M.Z., Murphy S., Zhao W., El-Amin I.B., Tan J., Dice D.D., Kang H.W., Jackson J.D., Yoo J.J., Atala A. (2012). In situ bioprinting of skin for reconstruction. J. Am. Coll. Surg..

[123] Hakimi N., Cheng R., Leng L., Sotoudehfar M., Ba P.Q., Bakhtyar N., Amini-Nik S., Jeschke M.G., Günther A. (2018). Handheld skin printer: In situ formation of planar biomaterials and tissues. Lab Chip.

[124] Bulanova E.A., Koudan E.V., Degosserie J., Heymans C., Pereira F.D.A.S., Parfenov V.A., Sun Y., Wang Q., Akhmedova S.A., Sviridova I.K. (2017). Bioprinting of a functional vascularized mouse thyroid gland construct. Biofabrication.

[125] Milovanovic I., Hayes M. (2018). Marine Gelatine from Rest Raw Materials. Appl. Sci. Basel.

[126] Ahmed R., Haq M., Chun B.S. (2019). Characterization of marine derived collagen extracted from the by-products of bigeye tuna (Thunnus obesus). Int. J. Biol. Macromol..

[127] Subhan F., Ikram M., Shehzad A., Ghafoor A. (2015). Marine Collagen: An Emerging Player in Biomedical applications. J. Food Sci. Technol..

[128] Wang L., An X., Yang F., Xin Z., Zhao L., Hu Q. (2008). Isolation and characterisation of collagens from the skin, scale and bone of deep-sea redfish (Sebastes mentella). Food Chem..

[129] Cho J.K., Jin Y.G., Rha S.J., Kim S.J., Hwang J.H. (2014). Biochemical characteristics of four marine fish skins in Korea. Food Chem..

[130] Wang H., Liang Y., Wang H., Zhang H., Wang M., Liu L. (2014). Physical-Chemical Properties of Collagens from Skin, Scale, and Bone of Grass Carp (Ctenopharyngodon idellus). J. Aquat. Food Prod. Technol..

[131] Rahman M.A. (2019). Collagen of Extracellular Matrix from Marine Invertebrates and Its Medical Applications. Mar. Drugs.

[132] Suntornnond R., An J., Chua C.K. (2017). Bioprinting of Thermoresponsive Hydrogels for Next Generation Tissue Engineering: A Review. Macromol. Mater. Eng..

[133] Xu W., Wang X., Yan Y., Zheng W., Xiong Z., Lin F., Wu R., Zhang R. (2007). Rapid Prototyping Three-Dimensional Cell/Gelatin/Fibrinogen Constructs for Medical Regeneration. J. Bioact. Compat. Polym..

[134] Wang X., Yan Y., Pan Y., Xiong Z., Liu H., Cheng J., Liu F., Lin F., Wu R., Zhang R. (2006). Generation of Three-Dimensional Hepatocyte/Gelatin Structures with Rapid Prototyping System. Tissue Eng..

[135] Yan Y., Wang X., Xiong Z., Liu H., Liu F., Lin F., Wu R., Zhang R., Lu Q. (2005). Direct Construction of a Three-dimensional Structure with Cells and Hydrogel. J. Bioact. Compat. Polym..

[136] Chung J.H.Y., Naficy S., Yue Z., Kapsa R., Quigley A., Moulton S.E., Wallace G.G. (2013). Bio-ink properties and printability for extrusion printing living cells. Biomater. Sci..

[137] Bertassoni L.E., Cecconi M., Manoharan V., Nikkhah M., Hjortnaes J., Cristino A.L., Barabaschi G., Demarchi D., Dokmeci M.R., Yang Y. (2014). Hydrogel bioprinted microchannel networks for vascularization of tissue engineering constructs. Lab Chip.

[138] Hoch E., Hirth T., Tovar G.E.M., Borchers K. (2013). Chemical tailoring of gelatin to adjust its chemical and physical properties for functional bioprinting. J. Mater. Chem. B.

[139] Ovsianikov A., Mühleder S., Torgersen J., Li Z., Qin X.H., Van Vlierberghe S., Dubruel P., Holnthoner W., Redl H., Liska R. (2014). Laser Photofabrication of Cell-Containing Hydrogel Constructs. Langmuir.

[140] Gómez-Guillén M.C., Pérez-Mateos M., Gómez-Estaca J., López-Caballero E., Giménez B., Montero P. (2009). Fish gelatin: A renewable material for developing active biodegradable films. Trends Food Sci. Technol..

[141] Karim A.A., Bhat R. (2009). Fish gelatin: Properties, challenges, and prospects as an alternative to mammalian gelatins. Food Hydrocoll..

[142] Feng W., Feng S., Tang K., He X., Jing A., Liang G. (2017). A novel composite of collagen-hydroxyapatite/kappa-carrageenan. J. Alloys Compd..

[143] Li C., Wang K., Zhou X., Li T., Xu Y., Qiang L., Peng M., Xu Y., Xie L., He C. (2019). Controllable fabrication of hydroxybutyl chitosan/oxidized chondroitin sulfate hydrogels by 3D bioprinting technique for cartilage tissue engineering. Biomed. Mater..

[144] Yoon H.J., Shin S.R., Cha J.M., Lee S.H., Kim J.H., Do J.T., Song H., Bae H. (2016). Cold Water Fish Gelatin Methacryloyl Hydrogel for Tissue Engineering Application. PLoS ONE.

[145] Schäffler A., Büchler C. (2007). Concise Review: Adipose Tissue-Derived Stromal Cells—Basic and Clinical Implications for Novel Cell-Based Therapies. Stem Cells.

[146] Gruene M., Pflaum M., Deiwick A., Koch L., Schlie S., Unger C., Wilhelmi M., Haverich A., Chichkov B.N. (2011). Adipogenic differentiation of laser-printed 3D tissue grafts consisting of human adipose-derived stem cells. Biofabrication.

[147] Butcher J.T., Mahler G.J., Hockaday L.A. (2011). Aortic valve disease and treatment: The need for naturally engineered solutions. Adv. Drug Deliv. Rev..

[148] Kang L.H., Armstrong P.A., Lee L.J., Duan B., Kang K.H., Butcher J.T. (2017). Optimizing Photo-Encapsulation Viability of Heart Valve Cell Types in 3D Printable Composite Hydrogels. Ann. Biomed. Eng..

[149] Loozen L.D., Wegman F., Öner F.C., Dhert W.J.A., Alblas J. (2013). Porous bioprinted constructs in BMP-2 non-viral gene therapy for bone tissue engineering. J. Mater. Chem. B.

[150] Muller W.E.G., Schroder H.C., Feng Q.L., Schlossmacher U., Link T., Wang X.H. (2015). Development of a morphogenetically active scaffold for three-dimensional growth of bone cells: Biosilica-alginate hydrogel for SaOS-2 cell cultivation. J. Tissue Eng. Regen. Med..

[151] Narayanan L.K., Huebner P., Fisher M.B., Spang J.T., Starly B., Shirwaiker R.A. (2016). 3D-Bioprinting of Polylactic Acid (PLA) Nanofiber-Alginate Hydrogel Bioink Containing Human Adipose-Derived Stem Cells. ACS Biomater. Sci. Eng..

[152] Ahlfeld T., Cidonio G., Kilian D., Duin S., Akkineni A.R., Dawson J.I., Yang S., Lode A., Oreffo R.O.C., Gelinsky M. (2017). Development of a clay based bioink for 3D cell printing for skeletal application. Biofabrication.

[153] Bendtsen S.T., Quinnell S.P., Wei M. (2017). Development of a novel alginate-polyvinyl alcohol-hydroxyapatite hydrogel for 3D bioprinting bone tissue engineered scaffolds. J. Biomed. Mater. Res. Part A.

[154] Bendtsen S.T., Wei M. (2017). In vitro evaluation of 3D bioprinted tri-polymer network scaffolds for bone tissue regeneration. J. Biomed. Mater. Res. Part A.

[155] Cunniffe G.M., Gonzalez-Fernandez T., Daly A., Sathy B.N., Jeon O., Alsberg E., Kelly D.J. (2017). Three-Dimensional Bioprinting of Polycaprolactone Reinforced Gene Activated Bioinks for Bone Tissue Engineering. Tissue Eng. Part A.

[156] Ojansivu M., Rashad A., Ahlinder A., Massera J., Mishra A., Syverud K., Finne-Wistrand A., Miettinen S., Mustafa K. (2019). Wood-based nanocellulose and bioactive glass modified gelatin–alginate bioinks for 3D bioprinting of bone cells. Biofabrication.

[157] da Conceicao Ribeiro R., Pal D., Ferreira A.M., Gentile P., Benning M., Dalgarno K. (2018). Reactive jet impingement bioprinting of high cell density gels for bone microtissue fabrication. Biofabrication.

[158] Daly A.C., Cunniffe G.M., Sathy B.N., Jeon O., Alsberg E., Kelly D.J. (2016). 3D Bioprinting of Developmentally Inspired Templates for Whole Bone Organ Engineering. Adv. Healthc. Mater..

[159] Izadifar M., Kelly M.E., Chen X. (2014). Engineering Angiogenesis for Myocardial Infarction Repair: Recent Developments, Challenges, and Future Directions. Cardiovasc. Eng. Technol..

[160] Zhang Y.S., Arneri A., Bersini S., Shin S.R., Zhu K., Goli-Malekabadi Z., Aleman J., Colosi C., Busignani F., Dell’Erba V. (2016). Bioprinting 3D microfibrous scaffolds for engineering endothelialized myocardium and heart-on-a-chip. Biomaterials.

[161] Izadifar M., Chapman D., Babyn P., Chen X., Kelly M.E. (2018). UV-Assisted 3D Bioprinting of Nanoreinforced Hybrid Cardiac Patch for Myocardial Tissue Engineering. Tissue Eng. Part C Methods.

[162] Maiullari F., Costantini M., Milan M., Pace V., Chirivì M., Maiullari S., Rainer A., Baci D., Marei H.E.S., Seliktar D. (2018). A multi-cellular 3D bioprinting approach for vascularized heart tissue engineering based on HUVECs and iPSC-derived cardiomyocytes. Sci. Rep..

[163] Izadifar M., Babyn P., Kelly M.E., Chapman D., Chen X. (2017). Bioprinting Pattern-Dependent Electrical/Mechanical Behavior of Cardiac Alginate Implants: Characterization and Ex Vivo Phase-Contrast Microtomography Assessment. Tissue Eng. Part C Methods.

[164] Kim S.W., Kim D.Y., Roh H.H., Kim H.S., Lee J.W., Lee K.Y. (2019). Three-Dimensional Bioprinting of Cell-Laden Constructs Using Polysaccharide-Based Self-Healing Hydrogels. Biomacromolecules.

[165] Shim J.H., Lee J.S., Kim J.Y., Cho D.W. (2012). Bioprinting of a mechanically enhanced three-dimensional dual cell-laden construct for osteochondral tissue engineering using a multi-head tissue/organ building system. J. Micromech. Microeng..

[166] Zhang Y., Yu Y., Chen H., Ozbolat I.T. (2013). Characterization of printable cellular micro-fluidic channels for tissue engineering. Biofabrication.

[167] Kundu J., Shim J.H., Jang J., Kim S.W., Cho D.W. (2015). An additive manufacturing-based PCL–alginate–chondrocyte bioprinted scaffold for cartilage tissue engineering. J. Tissue Eng. Regen. Med..

[168] Izadifar Z., Chang T.J., Kulyk W., Chen X.B., Eames B.F. (2016). Analyzing Biological Performance of 3D-Printed, Cell-Impregnated Hybrid Constructs for Cartilage Tissue Engineering. Tissue Eng. Part C Methods.

[169] Baena J., Jiménez G., López-Ruiz E., Antich C., Griñán-Lisón C., Perán M., Gálvez-Martín P., Marchal J. (2019). Volume-by-volume bioprinting of chondrocytes-alginate bioinks in high temperature thermoplastic scaffolds for cartilage regeneration. Exp. Biol. Med..

[170] Popa E., Reis R., Gomes M. (2012). Chondrogenic phenotype of different cells encapsulated in κ-carrageenan hydrogels for cartilage regeneration strategies. Biotechnol. Appl. Biochem..

[171] Martínez Ávila H., Schwarz S., Rotter N., Gatenholm P. (2016). 3D bioprinting of human chondrocyte-laden nanocellulose hydrogels for patient-specific auricular cartilage regeneration. Bioprinting.

[172] Müller M., Öztürk E., Arlov Ø., Gatenholm P., Zenobi-Wong M. (2017). Alginate Sulfate–Nanocellulose Bioinks for Cartilage Bioprinting Applications. Ann. Biomed. Eng..

[173] Hodder E., Duin S., Kilian D., Ahlfeld T., Seidel J., Nachtigall C., Bush P., Covill D., Gelinsky M., Lode A. (2019). Investigating the effect of sterilisation methods on the physical properties and cytocompatibility of methyl cellulose used in combination with alginate for 3D-bioplotting of chondrocytes. J. Mater. Sci. Mater. Med..

[174] You F., Chen X., Cooper D.M.L., Chang T., Eames B.F. (2018). Homogeneous hydroxyapatite/alginate composite hydrogel promotes calcified cartilage matrix deposition with potential for three-dimensional bioprinting. Biofabrication.

[175] Rathan S., Dejob L., Schipani R., Haffner B., Möbius M.E., Kelly D.J. (2019). Fiber Reinforced Cartilage ECM Functionalized Bioinks for Functional Cartilage Tissue Engineering. Adv. Healthc. Mater..

[176] Athirasala A., Tahayeri A., Thrivikraman G., França C.M., Monteiro N., Tran V., Ferracane J., Bertassoni L.E. (2018). A dentin-derived hydrogel bioink for 3D bioprinting of cell laden scaffolds for regenerative dentistry. Biofabrication.

[177] Yu H.Y., Zhang X.Y., Song W.J., Pan T., Wang H., Ning T.T., Wei Q., Xu H.H.K., Wu B.L., Ma D.D. (2019). Effects of 3-dimensional Bioprinting Alginate/Gelatin Hydrogel Scaffold Extract on Proliferation and Differentiation of Human Dental Pulp Stem Cells. J. Endod..

[178] Jeon H., Kang K., Park S.A., Kim W.D., Paik S.S., Lee S.H., Jeong J., Choi D. (2017). Generation of Multilayered 3D Structures of HepG2 Cells Using a Bio-printing Technique. Gut Liver.

[179] Kim Y., Kang K., Jeong J., Paik S.S., Kim J.S., Park S.A., Kim W.D., Park J., Choi D. (2017). Three-dimensional (3D) printing of mouse primary hepatocytes to generate 3D hepatic structure. Ann. Surg. Treat. Res..

[180] Kang K., Kim Y., Jeon H., Lee S.B., Kim J.S., Park S.A., Kim W.D., Yang H.M., Kim S.J., Jeong J. (2018). Three-Dimensional Bioprinting of Hepatic Structures with Directly Converted Hepatocyte-Like Cells. Tissue Eng. Part A.

[181] Hiller T., Berg J., Elomaa L., Roehrs V., Ullah I., Schaar K., Dietrich A.C., Al-Zeer M.A., Kurtz A., Hocke A.C. (2018). Generation of a 3D Liver Model Comprising Human Extracellular Matrix in an Alginate/Gelatin-Based Bioink by Extrusion Bioprinting for Infection and Transduction Studies. Int. J. Mol. Sci..

[182] Li X., Wang X., Wang X., Chen H., Zhang X., Zhou L., Xu T. (2018). 3D bioprinted rat Schwann cell-laden structures with shape flexibility and enhanced nerve growth factor expression. 3 Biotech.

[183] Ning L., Sun H., Lelong T., Guilloteau R., Zhu N., Schreyer D.J., Chen X. (2018). 3D bioprinting of scaffolds with living Schwann cells for potential nerve tissue engineering applications. Biofabrication.

[184] Gu Q., Tomaskovic-Crook E., Wallace G.G., Crook J.M., Chawla K. (2018). Engineering Human Neural Tissue by 3D Bioprinting. Biomaterials for Tissue Engineering: Methods and Protocols.

[185] Costantini M., Testa S., Mozetic P., Barbetta A., Fuoco C., Fornetti E., Tamiro F., Bernardini S., Jaroszewicz J., Święszkowski W. (2017). Microfluidic-enhanced 3D bioprinting of aligned myoblast-laden hydrogels leads to functionally organized myofibers in vitro and in vivo. Biomaterials.

[186] Mozetic P., Giannitelli S.M., Gori M., Trombetta M., Rainer A. (2017). Engineering muscle cell alignment through 3D bioprinting. J. Biomed. Mater. Res. Part A.

[187] Garcia-Lizarribar A., Fernandez-Garibay X., Velasco-Mallorqui F., Castano A.G., Samitier J., Ramon-Azcon J. (2018). Composite Biomaterials as Long-Lasting Scaffolds for 3D Bioprinting of Highly Aligned Muscle Tissue. Macromol. Biosci..

[188] Velasquillo C., Galue E.A., Rodriquez L., Ibarra C., Ibarraibarra L.G. (2013). Skin 3D Bioprinting. Applications in Cosmetology. J. Cosmet. Dermatol. Sci. Appl..

[189] Ding H., Chang R.C. (2018). Simulating image-guided in situ bioprinting of a skin graft onto a phantom burn wound bed. Addit. Manuf..

[190] Li J.L., Chi J.H., Liu J., Gao C., Wang K.X., Shan T., Li Y.Q., Shang W., Gu F. (2017). 3D printed gelatin-alginate bioactive scaffolds combined with mice bone marrow mesenchymal stem cells: A biocompatibility study. Int. J. Clin. Exp. Pathol..

[191] Pourchet L.J., Thepot A., Albouy M., Courtial E.J., Boher A., Blum L.J., Marquette C.A. (2017). Human Skin 3D Bioprinting Using Scaffold-Free Approach. Adv. Healthc. Mater..

[192] Fu X.B. (2005). Growth factors and skin repair and regeneration. Int. J. Cosmet. Sci..

[193] Huang S., Yao B., Xie J., Fu X. (2016). 3D bioprinted extracellular matrix mimics facilitate directed differentiation of epithelial progenitors for sweat gland regeneration. Acta Biomater..

[194] Gao Q., He Y., Fu J.Z., Liu A., Ma L. (2015). Coaxial nozzle-assisted 3D bioprinting with built-in microchannels for nutrients delivery. Biomaterials.

[195] Yu Y., Zhang Y., Martin J.A., Ozbolat I.T. (2013). Evaluation of Cell Viability and Functionality in Vessel-like Bioprintable Cell-Laden Tubular Channels. J. Biomech. Eng..

[196] Lee K.W., Kim D.H., Lee J.H., Youn Y.N. (2018). The Effect of Pulsatile Flow on bMSC-Derived Endothelial-Like Cells in a Small-Sized Artificial Vessel Made by 3-Dimensional Bioprinting. Stem Cells Int..

[197] Hewes S., Wong A.D., Searson P.C. (2017). Bioprinting microvessels using an inkjet printer. Bioprinting.

[198] Attalla R., Puersten E., Jain N., Selvaganapathy P.R. (2018). 3D bioprinting of heterogeneous bi- and tri-layered hollow channels within gel scaffolds using scalable multi-axial microfluidic extrusion nozzle. Biofabrication.

[199] Neufurth M., Wang X., Schröder H.C., Feng Q., Diehl-Seifert B., Ziebart T., Steffen R., Wang S., Müller W.E.G. (2014). Engineering a morphogenetically active hydrogel for bioprinting of bioartificial tissue derived from human osteoblast-like SaOS-2 cells. Biomaterials.

[200] Kesti M., Eberhardt C., Pagliccia G., Kenkel D., Grande D., Boss A., Zenobi-Wong M. (2015). Bioprinting Complex Cartilaginous Structures with Clinically Compliant Biomaterials. Adv. Funct. Mater..

[201] Costantini M., Idaszek J., Szöke K., Jaroszewicz J., Dentini M., Barbetta A., Brinchmann J.E., Święszkowski W. (2016). 3D bioprinting of BM-MSCs-loaded ECM biomimetic hydrogels forin vitroneocartilage formation. Biofabrication.

[202] Daly A.C., Critchley S.E., Rencsok E.M., Kelly D.J. (2016). A comparison of different bioinks for 3D bioprinting of fibrocartilage and hyaline cartilage. Biofabrication.

[203] Apelgren P., Amoroso M., Lindahl A., Brantsing C., Rotter N., Gatenholm P., Kolby L. (2017). Chondrocytes and stem cells in 3D-bioprinted structures create human cartilage in vivo. PLoS ONE.

[204] Apelgren P., Amoroso M., Saeljoe K., Lindahl A., Brantsing C., Stridh Orrhult L., Gatenholm P., Kolby L. (2018). Skin Grafting on 3D Bioprinted Cartilage Constructs In Vivo. Plast. Reconstr. Surg. Glob. Open.

[205] Kosik-Kozioł A., Costantini M., Bolek T., Szöke K., Barbetta A., Brinchmann J., Święszkowski W. (2017). PLA short sub-micron fiber reinforcement of 3D bioprinted alginate constructs for cartilage regeneration. Biofabrication.

[206] Nguyen D., Hägg D.A., Forsman A., Ekholm J., Nimkingratana P., Brantsing C., Kalogeropoulos T., Zaunz S., Concaro S., Brittberg M. (2017). Cartilage Tissue Engineering by the 3D Bioprinting of iPS Cells in a Nanocellulose/Alginate Bioink. Sci. Rep..

[207] Yang X., Lu Z., Wu H., Li W., Zheng L., Zhao J. (2018). Collagen-alginate as bioink for three-dimensional (3D) cell printing based cartilage tissue engineering. Mater. Sci. Eng. C.

[208] Chang R., Emami K., Wu H., Sun W. (2010). Biofabrication of a three-dimensional liver micro-organ as anin vitrodrug metabolism model. Biofabrication.

[209] Wu Y., Lin Z.Y., Wenger A.C., Tam K.C., Tang X. (2018). 3D bioprinting of liver-mimetic construct with alginate/cellulose nanocrystal hybrid bioink. Bioprinting.

[210] Gao Q., Liu Z.J., Lin Z.W., Qiu J.J., Liu Y., Liu A., Wang Y.D., Xiang M.X., Chen B., Fu J.Z. (2017). 3D Bioprinting of Vessel-like Structures with Multilevel Fluidic Channels. ACS Biomater. Sci. Eng..

[211] Pi Q.M., Maharjan S., Yan X., Liu X., Singh B., van Genderen A.M., Robledo-Padilla F., Parra-Saldivar R., Hu N., Jia W.T. (2018). Digitally Tunable Microfluidic Bioprinting of Multilayered Cannular Tissues. Adv. Mater..

[212] Wilson S.A., Cross L.M., Peak C.W., Gaharwar A.K. (2017). Shear-Thinning and Thermo-Reversible Nanoengineered Inks for 3D Bioprinting. ACS Appl. Mater. Interfaces.

[213] Chimene D., Peak C.W., Gentry J.L., Carrow J.K., Cross L.M., Mondragon E., Cardoso G.B., Kaunas R., Gaharwar A.K. (2018). Nanoengineered Ionic–Covalent Entanglement (NICE) Bioinks for 3D Bioprinting. ACS Appl. Mater. Interfaces.

[214] Huang J., Fu H., Wang Z., Meng Q., Liu S., Wang H., Zheng X., Dai J., Zhang Z. (2016). BMSCs-laden gelatin/sodium alginate/carboxymethyl chitosan hydrogel for 3D bioprinting. RSC Adv..

[215] Marchioli G., van Gurp L., van Krieken P.P., Stamatialis D., Engelse M., van Blitterswijk C.A., Karperien M.B.J., de Koning E., Alblas J., Moroni L. (2015). Fabrication of three-dimensional bioplotted hydrogel scaffolds for islets of Langerhans transplantation. Biofabrication.

[216] Liu X., Carter S.S.D., Renes M.J., Kim J., Rojas-Canales D.M., Penko D., Angus C., Beirne S., Drogemuller C.J., Yue Z. (2019). Development of a Coaxial 3D Printing Platform for Biofabrication of Implantable Islet-Containing Constructs. Adv. Healthc. Mater..

[217] Duin S., Schütz K., Ahlfeld T., Lehmann S., Lode A., Ludwig B., Gelinsky M. (2019). 3D Bioprinting of Functional Islets of Langerhans in an Alginate/Methylcellulose Hydrogel Blend. Adv. Healthc. Mater..

[218] Irvine S.A., Venkatraman S.S. (2016). Bioprinting and Differentiation of Stem Cells. Molecules.

[219] Lindsay C.D., Roth J.G., LeSavage B.L., Heilshorn S.C. (2019). Bioprinting of stem cell expansion lattices. Acta Biomater..

[220] Fang Y., Eglen R.M. (2017). Three-Dimensional Cell Cultures in Drug Discovery and Development. SLAS Discov. Adv. Life Sci. R D.

[221] Ma X., Qu X., Zhu W., Li Y.S., Yuan S., Zhang H., Liu J., Wang P., Lai C.S.E., Zanella F. (2016). Deterministically patterned biomimetic human iPSC-derived hepatic model via rapid 3D bioprinting. Proc. Natl. Acad. Sci. USA.

[222] Kang L., Chung B.G., Langer R., Khademhosseini A. (2008). Microfluidics for drug discovery and development: From target selection to product lifecycle management. Drug Discov. Today.

[223] Mihaela L. (2012). Tumor microenvironment in the brain. Cancers.

[224] Wang X., Dai X., Zhang X., Li X., Xu T., Lan Q. (2018). Enrichment of glioma stem cell-like cells on 3D porous scaffolds composed of different extracellular matrix. Biochem. Biophys. Res. Commun..

[225] Xingliang D., Cheng M., Qing L., Tao X. (2016). 3D bioprinted glioma stem cells for brain tumor model and applications of drug susceptibility. Biofabrication.

[226] Wang X., Li X., Dai X., Zhang X., Zhang J., Xu T., Lan Q. (2018). Coaxial extrusion bioprinted shell-core hydrogel microfibers mimic glioma microenvironment and enhance the drug resistance of cancer cells. Colloids Surf. B Biointerfaces.

[227] Kevin D.R., Sundararajan V.M. (2018). Bioprinted chitosan-gelatin thermosensitive hydrogels using an inexpensive 3D printer. Biofabrication.

[228] Vargo-Gogola T., Rosen J.M. (2007). Modelling breast cancer: One size does not fit all. Nat. Rev. Cancer.

[229] Wang X., Zhang X., Dai X., Wang X., Li X., Diao J., Xu T. (2018). Tumor-like lung cancer model based on 3D bioprinting. 3 Biotech.

[230] Jiang T., Munguia-Lopez J.G., Flores-Torres S., Grant J., Vijayakumar S., Leon-Rodriguez A.D., Kinsella J.M. (2017). Directing the Self-assembly of Tumour Spheroids by Bioprinting Cellular Heterogeneous Models within Alginate/Gelatin Hydrogels. Sci. Rep..

[231] Zhao Y., Yao R., Ouyang L., Ding H., Zhang T., Zhang K., Cheng S., Sun W. (2014). Three-dimensional printing of Hela cells for cervical tumor modelin vitro. Biofabrication.

[232] Swaminathan S., Hamid Q., Sun W., Clyne A.M. (2019). Bioprinting of 3D breast epithelial spheroids for human cancer models. Biofabrication.

[233] Diao J., Zhang C., Zhang D., Wang X., Zhang J., Ma C., Deng K., Jiang T., Jia W., Xu T. (2019). Role and mechanisms of a three-dimensional bioprinted microtissue model in promoting proliferation and invasion of growth-hormone-secreting pituitary adenoma cells. Biofabrication.

[234] Dai X., Liu L., Ouyang J., Li X., Zhang X., Lan Q., Xu T. (2017). Coaxial 3D bioprinting of self-assembled multicellular heterogeneous tumor fibers. Sci. Rep..

[235] Grigoryan B., Paulsen S.J., Corbett D.C., Sazer D.W., Fortin C.L., Zaita A.J., Greenfield P.T., Calafat N.J., Gounley J.P., Ta A.H. (2019). Multivascular networks and functional intravascular topologies within biocompatible hydrogels. Science.

[236] Mandrycky C., Wang Z., Kim K., Kim D.H. (2016). 3D bioprinting for engineering complex tissues. Biotechnol. Adv..

[237] Rinaudo M. (2008). Main properties and current applications of some polysaccharides as biomaterials. Polym. Int..

[238] Peppas N.A., Hilt J.Z., Khademhosseini A., Langer R. (2006). Hydrogels in Biology and Medicine: From Molecular Principles to Bionanotechnology. Adv. Mater..

[239] Noor N., Shapira A., Edri R., Gal I., Wertheim L., Dvir T. (2019). 3D Printing of Personalized Thick and Perfusable Cardiac Patches and Hearts. Adv. Sci..

[240] Wang X., Ao Q., Tian X., Fan J., Wei Y., Hou W., Tong H., Bai S. (2016). 3D Bioprinting Technologies for Hard Tissue and Organ Engineering. Materials.

[241] Jesionowski T., Norman M., Zoltowska-Aksamitowska S., Petrenko I., Joseph Y., Ehrlich H. (2018). Marine Spongin: Naturally Prefabricated 3D Scaffold-Based Biomaterial. Mar. Drugs.

[242] Lee J.S., Hong J.M., Jung J.W., Shim J.H., Oh J.H., Cho D.W. (2014). 3D printing of composite tissue with complex shape applied to ear regeneration. Biofabrication.

